# Complete mitochondrial genome of *Ficus hirta* and its comparative analysis

**DOI:** 10.3389/fgene.2025.1530105

**Published:** 2025-04-23

**Authors:** Wangdong Deng, Xinyi Cai

**Affiliations:** ^1^ Department of Urology, Longgang District Central Hospital of Shenzhen, Shenzhen, China; ^2^ Shenzhen Clinical School of Medicine, Guangzhou University of Chinese Medicine, Guangzhou, Guangdong, China; ^3^ College of Medicine, Shantou University, Shantou, China; ^4^ Department of Pathology, Shantou University Medical College, Shantou, China

**Keywords:** mitochondrial genomes, *Ficus hirta*, plant genomics, ecosystem, comparative analysis

## Abstract

**Introduction:**

*Ficus hirta*, known as Wuzhimaotao in China, is a dioecious plant species within the Moraceae family, highly regarded for its medicinal and ecological roles.

**Methods:**

Utilizing a hybrid assembly methodology, combining Nanopore and Illumina sequencing, we achieved a detailed mitochondrial genomic architecture.

**Results:**

The genome exhibits single circular structures, spans 486,226 base pairs with 45.21% GC content, and encompasses 31 distinct protein-coding genes.Our analysis extends to gene content, codon usage, intergenomic transfers, phylogenetic relationships, and RNA editing patterns. Notably, the mitochondrial and chloroplast genomes share 15 homologous fragments, underpinning intercellular gene exchange. Phylogenetic positioning confirms *Ficus hirta* within Moraceae, closely allied with *Morus notabilis*.

**Discussion:**

This comprehensive mitochondrial genome elucidation not only augments the biological understanding of *Ficus hirta* but also enriches genomic resources for future research.

## 1 Introduction

*Ficus hirta*, known as Wuzhimaotao in China, is a dioecious plant species within the Moraceae family, highly regarded for its medicinal and ecological roles (Wu et al., 2013; Yu et al., 2008; Sun et al., 2016). It has been extensively used in traditional herbal medicine, particularly in the Lingnan regions of China, with its dry root, Radix Fici Hirtae, being utilized for therapeutic purposes. Key bioactive compounds have been identified as the main active ingredients contributing to its anti-inflammatory and antioxidant effects (Cheng et al., 2017; Chen et al., 2020).

Ecologically, *F. hirta* is integral to its ecosystem, participating in intricate mutualisms. It depends on species-specific fig wasps for pollination, while vertebrates, including birds and bats, facilitate seed dispersal, emphasizing its role in promoting local biodiversity (Yu et al., 2010). Due to overharvesting driven by market demand for its medicinal properties, wild populations have significantly declined, raising concerns for its long-term survival. Despite this, genetic studies have revealed high genetic diversity and low population differentiation, indicating that *F. hirta* populations still have the resilience needed for conservation efforts and potential sustainable breeding programs (Lu et al., 2022).

Although much is known about the medicinal properties and chloroplast genome of *F. hirta*, its mitochondrial genome remains largely unexplored (Liu et al., 2019). Mitochondrial genomes are essential for understanding key aspects of plant energy metabolism, phylogenetic relationships, and genomic architecture. Recent advancements in sequencing technologies, particularly hybrid assembly methods combining long-read and short-read data, have enabled comprehensive analyses of plant mitochondrial genomes. This opens new avenues to investigate gene content, codon usage, intergenomic transfers, and evolutionary dynamics, making the study of *F. hirta*’s mitochondrial genome particularly significant in expanding our understanding of the species at the genomic level.

This study presents the first complete mitochondrial genome of *F. hirta*, alongside a comparative analysis with other species in the Rosales order. The study sheds light on intergenomic transfers between mitochondrial and chloroplast genomes, offering valuable insights into their evolutionary interactions. By analyzing phylogenetic relationships within the Moraceae family, this research enhances our understanding of *F. hirta*’s genomic framework, contributing to broader knowledge about the evolutionary divergence of species within the Rosales order. The findings serve as an important resource for future conservation strategies and genomic studies of this ecologically and pharmacologically important species.

## 2 Materials and methods

### 2.1 Plant material and DNA extraction

Fresh leaves of *F. hirta* were collected from multiple locations in the Lingnan region of China. Total genomic DNA was extracted from approximately 100 mg of fresh leaf tissue using the cetyltrimethylammonium bromide (CTAB) method (Doyle and Doyle, 1987). DNA quantity and quality were assessed using a NanoDrop spectrophotometer and agarose gel electrophoresis (Wilfinger et al., 1997). High-quality DNA samples were selected for sequencing.

### 2.2 Sequencing and genome assembly

We employed a hybrid sequencing strategy using Illumina NovaSeq (150-bp paired-end reads, ∼5 Gb) and Nanopore PromethION (∼10 Gb of long-read data). Long-read assembly was first performed using Flye (v2.9) with default parameters, generating a graph-based assembly in GFA format. To identify mitochondrial contigs, we built a local BLAST database using makeblastdb (BLAST + v2.11.0) and conducted a BLASTn search against conserved mitochondrial genes from *Arabidopsis thaliana*, using parameters: evalue 1e-5 -outfmt 6 -max_hsps 10 -word_size 7 -task blastn-short. The identified mitochondrial contigs were visualized using Bandage (v0.8.1) for manual selection. Different mapping software and parameters for short-read and long-read sequencing data were used. For the short-read sequencing data, the paired-end sequencing reads were aligned to the reference genome using a multi-step mapping pipeline. First, the BWA-MEM algorithm (v0.7.17) was employed to generate genome indices with the command “bwa index”. Subsequent read alignment was performed using “bwa mem” with 10 computational threads (-t 10), processing both forward and reverse read files. The SAM output was piped to samtools (v1.15.1) for binary conversion (-bS) while filtering out unmapped reads (-F 12) using eight parallel threads (-@ 8). Reads were then name-sorted (sort -n) to maintain proper pairing information before being converted back to FASTQ format. The final aligned read pairs were output through samtools’ fastq module with thread-optimized processing (-@ 8). For the long-read sequencing data, Long-read sequencing data processing was performed through an optimized alignment pipeline. The reference genome was initially indexed using minimap2 (v2.24) with the command “minimap2 -d ref1.mmi”. Sequence alignment was executed using the Nanopore-preset parameters (-ax map-ont) with eight computational threads (-t 8), processing compressed long-read data (ont.fastq.gz) through “minimap2 -ax map-pb -t 8 ref1.mmi ont.fastq.gz > out.sam”. The resulting SAM file was converted to BAM format using samtools (v1.15.1) with unmapped read filtering (-F 4) and 8-thread parallelization (-@ 8). Final FASTQ generation was accomplished through bedtools’ (v2.30.0) bamtofastq module, producing the processed long-read dataset. The final hybrid assembly was performed using Unicycler (v0.4.8) with default parameters, producing a circular mitochondrial genome, which was confirmed and visualized again using Bandage (v0.8.1). The final mitochondrial genome assembly was polished using Illumina short-read data with Pilon (v1.23) to improve assembly accuracy.

### 2.3 Genome annotation and analysis

The annotation of the mitochondrial genome was performed using GeSeq (v1.85), with *A. thaliana* and Liriodendron tulipifera mitochondrial genomes used as reference sequences. We chose *A. thaliana* and Liriodendron tulipifera as references for gene annotation because they are well-annotated model plants with high-quality mitochondrial annotation covering typical angiosperm gene sets, which widely used for mitochondrial reference. tRNAscan-SE (v2.0) was employed for the identification of tRNA genes, while BLASTN **(v2.11.0)** was used for rRNA annotation. The annotations were manually curated using the Apollo software (v2.6.6) to ensure accuracy. The complete circular genome map was visualized using OGDRAW (v1.3.1) to represent gene locations and orientations.

### 2.4 Codon usage and repeat sequence analysis

Relative synonymous codon usage (RSCU) for all protein-coding genes (PCGs) was calculated using MEGA (v7.0.26). To assess repeat structures within the genome, MISA-web (v2.1) was used to identify simple sequence repeats (SSRs), and REPuter (v2.74) was employed to detect dispersed repeats, including forward, palindromic, and complementary repeats. Tandem repeat sequences were identified using Tandem Repeat Finder (TRF, v4.09) 81% as matching-score threshold for tandem repeats. This threshold is a commonly used default in Tandem Repeat Finder for moderate stringency, balancing sensitivity and specificity. And all repeat sequence results were visualized using Circos (v0.69–9).

### 2.5 Intergenomic transfer and phylogenetic analysis

Homologous regions between the mitochondrial and chloroplast genomes were identified using BLASTN (v2.11.0) with a minimum alignment length of 100 bp and identity greater than 90%. The sequence transfer between the two genomes was further analyzed and visualized using Circos (v0.69-9). The chloroplast genome was assembled using GetOrganelle software (v1.7.7.0) (Jin et al., 2020), annotated using CPGAVAS2 software (Shi et al., 2019), and then corrected using CPGView software (Liu et al., 2023). The rrn16S, rrn23S, rrn4.5S, and rrn5S genes were identified based on sequence homology and conserved domain searches. Subsequently, we masked or removed the corresponding regions from further analyses to prevent potential biases introduced by highly conserved sequences. This methodological approach aligns with previous studies emphasizing the necessity of removing highly conserved organellar genes to improve the accuracy of horizontal gene transfer detection (Mower et al., 2010; Timmis et al., 2004). Phylogenetic analysis was performed using MAFFT (v7.475) for multiple sequence alignment and IQ-TREE (v2.1.4) for maximum likelihood analysis. Morus notabilis and other closely related species in the Moraceae family were included for comparative analysis The phylogenetic tree included 31 species across multiple families. Rosaceae species included *Pyrus × bretschneideri* (NC_065218.1), *Pyrus betulifolia* (NC_054332.1), *Pyrus communis* (NC_065229.1), *Eriobotrya japonica* (NC_045228.1), *Sorbus aucuparia* (NC_052880.1), *Malus sylvestris* (NC_065226.1), *Malus sieversii* (NC_065225.1), *Chaenomeles speciosa* (OL450370.1), *Torminalis glaberrima* (MT610102.1), *Photinia serratifolia* (NC_065220.1), *Malus baccata* (NC_065224.1), *Prunus armeniaca* (NC_065228.1), *Prunus mira* (NC_065231.1), *Prunus kanzakura* (NC_065230.1), *Fragaria iturupensis* (NC_062833.1), *Fragaria gracilis* (NC_062834.1), *Fragaria vesca* (NC_065239.1), *Potentilla anserina* (ON478170.1), *Potentilla micrantha* (NC_062588.1), *Rosa rugosa* (NC_065237.1), *Rosa chinensis* (NC_065236.1), *Rubus chingii* (NC_065238.1), and *Geum urbanum* (NC_065221.1). Moraceae species included *F. hirta* (This Research) and *Morus notabilis* (NC_041177.1). Cannabaceae species included *Cannabis sativa* (NC_029855.1) and *Humulus lupulus* (MW413894.1). Rhamnaceae species included *Ziziphus mauritiana* (NC_068745.1) and *Ziziphus jujuba* (NC_029809.1). Fabaceae species included *Glycine max* (NC_020455.1), *Senna tora* (NC_038053.1).

### 2.6 RNA editing prediction

RNA editing sites in the mitochondrial genome of *F. hirta* were predicted using the PREP-Mt tool (v0.9), which is specifically designed to identify RNA editing events in plant mitochondrial genomes. The tool predicts C-to-U conversions, the most common form of RNA editing in plant mitochondria. The sequences of all 31 protein-coding genes from the *F. hirta* mitochondrial genome were input into the program. The cutoff value for editing prediction was set to 0.9, following standard parameters for accurate detection of potential RNA editing sites in plant mitochondrial genomes.

### 2.7 Synteny analysis

Synteny analysis was performed to compare the *F. hirta* mitochondrial genome with those of related species. Homologous regions between the mitochondrial genomes of *F. hirta* and other species were identified using the BLASTN tool (v2.11.0), with a minimum alignment length of 500 bp and sequence identity greater than 90%. MCScanX (v1.0) was used to detect and analyze syntenic blocks, including regions of conservation and rearrangement. The syntenic relationships were visualized using Circos (v0.69-9), providing a comprehensive view of genome structure variation among the species.

## 3 Results

### 3.1 Complete mitogenome sequence of *Ficus hirta*

**FIGURE 1:**
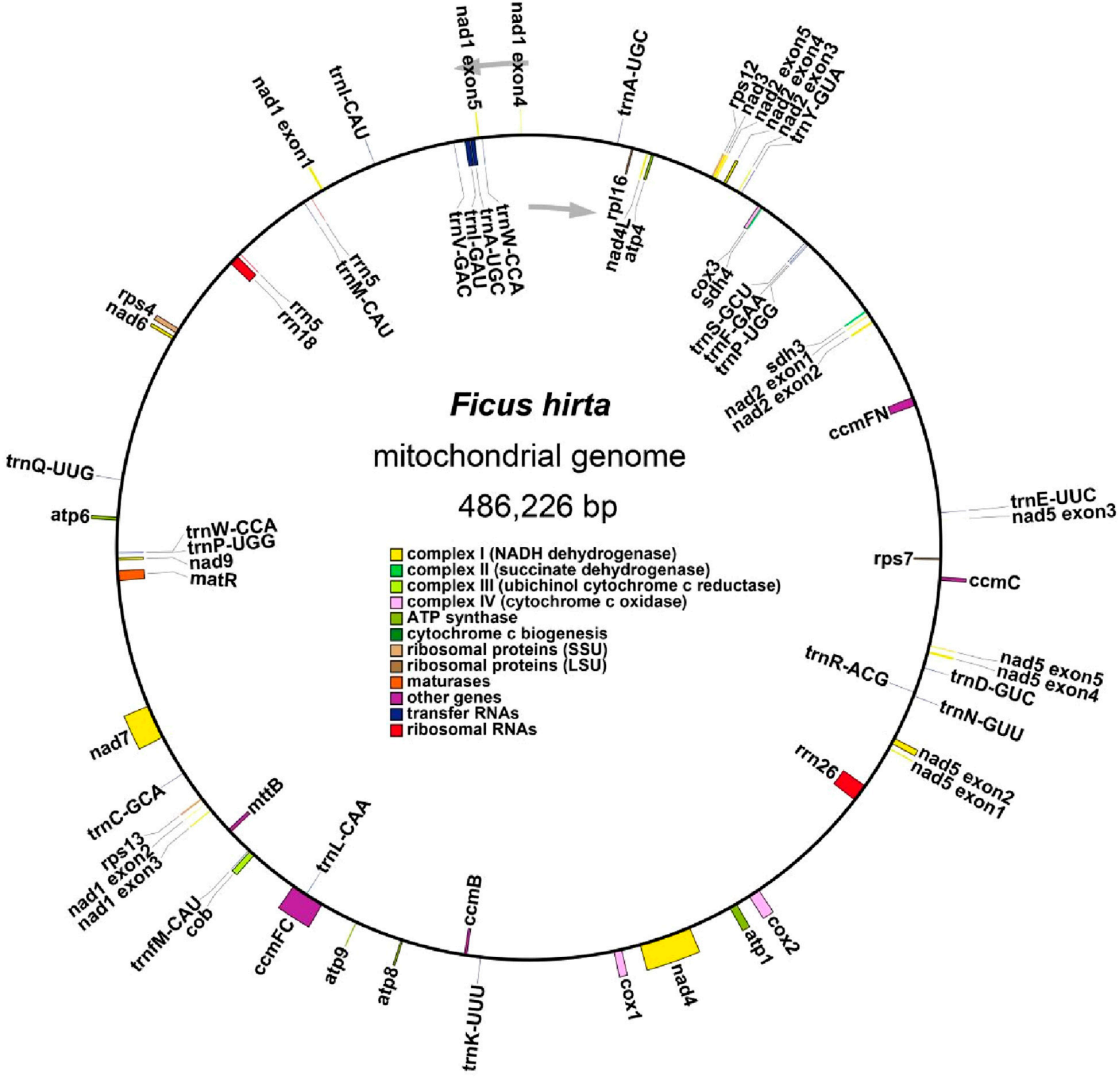
Detailed mitogenome map of *Ficus hirta* Colored boxes denote conserved mitochondrial genes categorized according to their functional products. Genes located inside the circle are transcribed in a clockwise direction, whereas those positioned outside the circle are transcribed in a counterclockwise direction. Genes associated with various functional categories are designated by distinct colors.

The mitochondrial genome of *F. hirta* is organized as a single circular molecule (Figure 1). The genome spans 486,226 base pairs (bp) with a GC content of 45.21%, which is comparable to other plant mitochondrial genomes within the Rosales order (Zhang et al., 2022). Annotation of the *F. hirta* mitochondrial genome identified 31 protein-coding genes, 19 tRNA genes, and three rRNA genes, with the tRNA and rRNA genes containing multi-copy variants, as shown in Supplementary Table S1. The protein-coding genes include 24 core mitochondrial genes and seven non-core genes, following the conserved gene set typically observed in plant mitochondrial genomes. Comparative analysis with *A. thaliana* and Liriodendron tulipifera confirmed the presence of all core genes, indicating a high level of conservation. Additionally, no novel protein-coding genes specific to *F. hirta* were identified, further supporting the evolutionary stability of plant mitochondrial genomes. The core genes include 5 ATP synthase genes (*atp1*, *atp4*, *atp6*, *atp8*, and *atp9*); 9 NADH dehydrogenase genes (*nad1*, *nad2*, *nad3*, *nad4*, *nad4L*, *nad5*, *nad6*, *nad7*, and *nad9*); four cytochrome *c* biogenesis genes (*ccmB*, *ccmC*, *ccmFC*, and *ccmFN*); three cytochrome *c* oxidase genes (*cox1*, *cox2*, and *cox3*); one protein transport subunit gene (*mttB*); one maturases gene (*matR*); and one cytochrome *b* gene (*cob*). The non-core genes include one ribosomal protein large subunit gene (*rpl16*); four ribosomal protein small subunit genes (*rps4*, *rps7*, *rps12*, *rps13*); and two succinate dehydrogenase genes (*sdh3*, *sdh4*). Details of genome assembly and gene annotations could be found in Supplementary Data Sheet 1.

### 3.2 Codon usage analysis of protein-coding genes (PCGs) in *Ficus hirta*s

**FIGURE 2:**
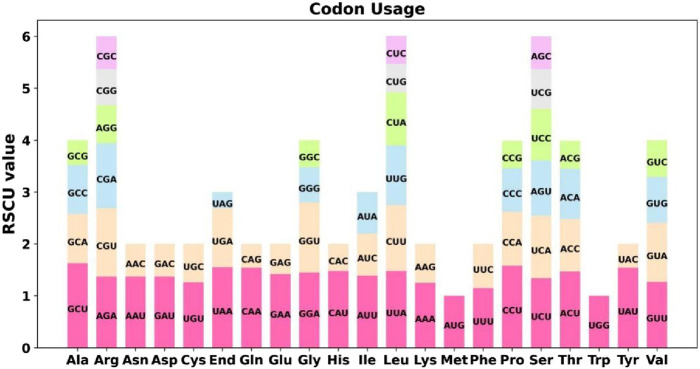
Codon Usage Analysis of the *Ficus hirta* Mitochondrial Genome. X-axis, codon families; Y-axis, the RSCU value.

Codon usage analysis was conducted on the 31 unique PCGs of the *Ficus hirta*s mitochondrial genome (Figure 2; Supplementary Table S2). The codon usage for each amino acid is presented in Supplementary Table S2. Codons with a relative synonymous codon usage (RSCU) greater than one are considered preferentially used by amino acids. As shown in Figure 2, apart from the start codon AUG and tryptophan (UGG), both of which have an RSCU value of 1, the PCGs of the mitochondrial genome exhibit a general codon usage bias. For example, alanine (Ala) shows a strong preference for GCU, with the highest RSCU value of 1.63 among mitochondrial PCGs, followed by proline (Pro), which preferentially uses CCU, with an RSCU value of 1.58. Notably, phenylalanine (Phe) has a maximum RSCU value of less than 1.2, indicating a relatively weak codon usage bias.

### 3.3 Repeat sequence analysis on the *Ficus hirta* mitochondrial genome

**FIGURE 3:**
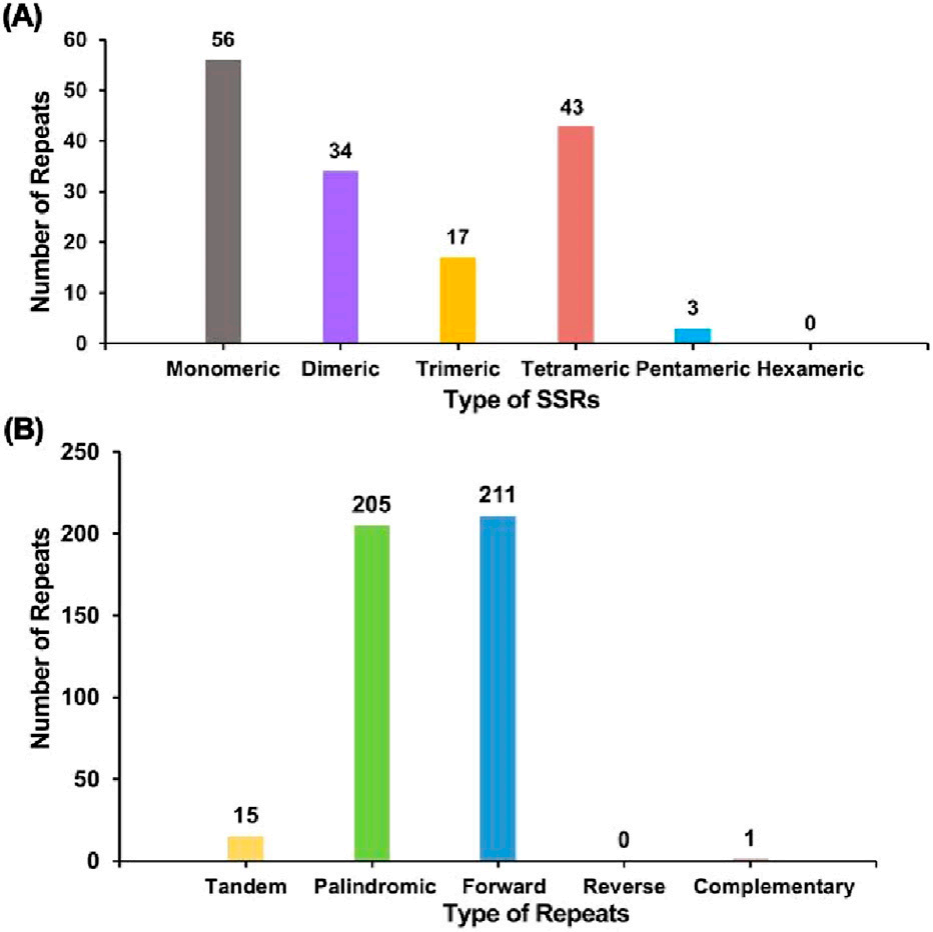
Repeat sequence analysis on the *Ficus hirta* mitochondrial genome. **(A)** The x-axis represents the types of SSRs, and the y-axis indicates the number of repeat fragments. The gray legend represents mononucleotide SSRs, the purple legend represents dinucleotide SSRs, the yellow legend represents trinucleotide SSRs, the red legend represents tetranucleotide SSRs, and the blue legend represents pentanucleotide SSRs. No hexanucleotide SSRs were detected in this mitochondrial genome. **(B)** The x-axis represents the types of repeat sequences, and the y-axis indicates the number of repeat fragments. The yellow legend represents tandem repeats, the green legend represents palindromic repeats, the blue legend represents forward repeats, and the red legend represents complementary repeats. No reverse repeats were detected in this mitochondrial genome.

A repeat sequence analysis was performed on the *F. hirta* mitochondrial genome (Figure 3; Supplementary Tables S3–S5). A total of 153 SSRs were detected, with mononucleotide and dinucleotide SSRs accounting for 58.82% of the total SSRs, and mononucleotide adenine repeats making up 53.57% of mononucleotide SSRs. No hexanucleotide SSRs were observed. Tandem repeats, which are core repeat units ranging from 7 to 200 bp, were also identified, with 15 tandem repeats found in the *F. hirta* mitochondrial genome, exhibiting a matching score greater than 81% and lengths varying from 15 to 48 bp.

Additionally, 417 dispersed repeats longer than 30 bp were detected, comprising 205 palindromic repeats, 211 forward repeats, and one complementary repeat, with no reverse repeats observed. The longest palindromic repeat extended 12,060 bp, while the longest forward repeat measured 521 bp. These repeat sequences were distributed across the mitochondrial genome, primarily occurring within intergenic regions, which is consistent with findings in other plant mitochondrial genomes (Fukasawa et al., 2024; Zhong et al., 2022; Wang et al., 2024).

### 3.4 Sequence transfer analysis of mitochondrial genome structure in *Ficus hirta*

**FIGURE 4:**
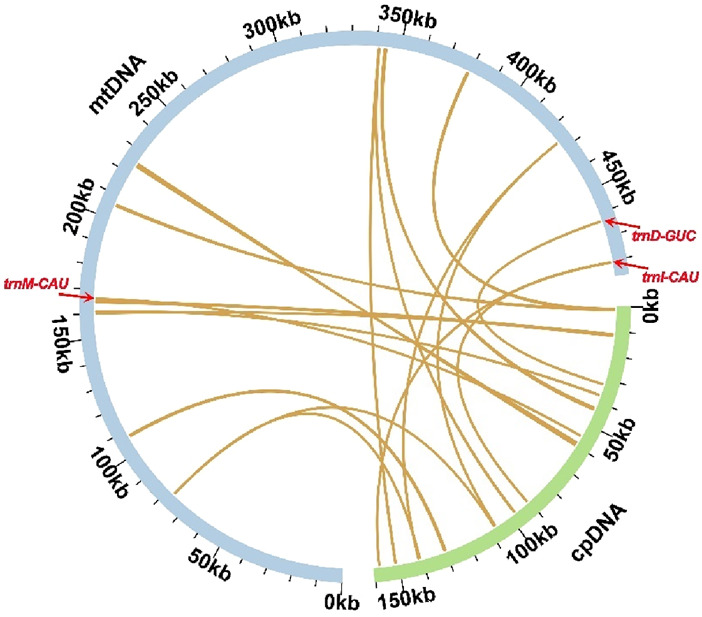
Sequence transfer analysis. Blue arcs represent the mitochondrial genome, while green arcs represent the chloroplast genome. The yellow lines between the arcs correspond to homologous genome segments. The homologous fragments containing complete tRNA genes (trnD-GUC, trnI-CAU, and trnM-CAU) were distinctly marked.

Based on sequence similarity analysis, a total of 15 segments were identified as homologous between the mitochondrial and chloroplast genomes, with a combined length of 4,979 bp, accounting for 1.02% of the mitochondrial genome. The longest segment, MTPT7, spans 1,266 bp. Annotation of these homologous sequences revealed three complete genes, all of which are tRNA genes (trnD-GUC, trnI-CAU, trnM-CAU), located within the 15 homologous segments (Figure 4; Supplementary Table S6 and Supplementary Data Sheet 2).

### 3.5 phylogenetic analysis of mitochondrial genome structure in *Ficus hirta*

**FIGURE 5:**
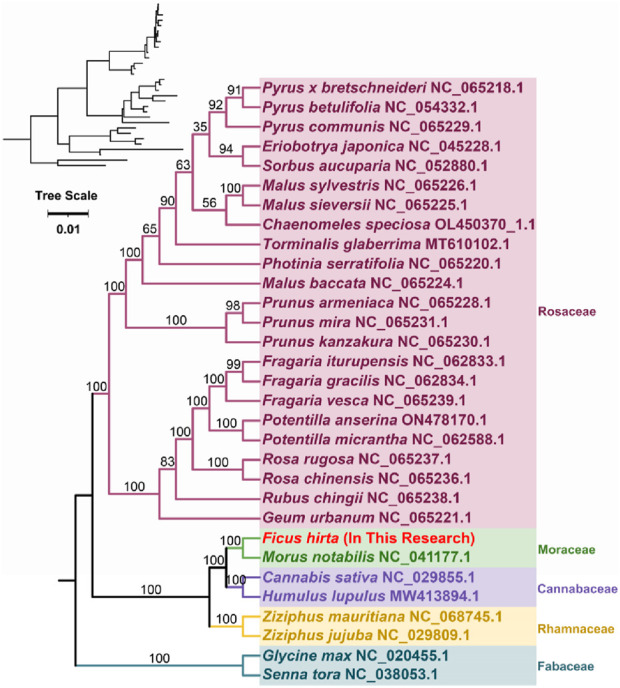
Phylogenetic analysis was conducted based on the DNA sequences of 23 conserved mitochondrial protein-coding genes (PCGs). The tree scale represents the number of nucleotide substitutions per site. The bootstrap values are indicated at the nodes.

Phylogenetic analysis was conducted based on the DNA sequences of 23 conserved mitochondrial protein-coding genes (PCGs) (Figure 5). A phylogenetic tree was constructed for 31 species across five families of angiosperms. The shared protein-coding genes include *atp1*, *atp4*, *atp6*, *atp8*, *atp9*, *ccmB*, *ccmC*, *ccmFC*, *ccmFN*, *cob*, *cox1*, *cox2*, *cox3*, *matR*, *mttB*, *nad2*, *nad3*, *nad4*, *nad4L*, *nad5*, *nad6*, *nad7*, and *nad9*. Two mitochondrial genomes from the Fabaceae family were used as outgroups. The phylogenetic topology based on mitochondrial DNA aligns with the most recent classification by the Angiosperm Phylogeny Group (APG) (Tap et al., 2016). Species *F. hirta* belongs to the order Rosales, family Moraceae, and is closely related to Morus notabilis (Chinese mulberry).

### 3.6 Analysis of RNA editing events of mitochondrial genome structure in *Ficus hirta*

**FIGURE 6:**
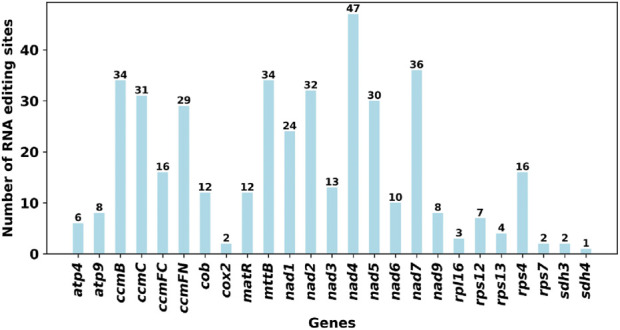
The Predicted Number of RNA Editing Sites in Mitochondrial PCGs, which focused on predicted C-to-U RNA editing sites.

**FIGURE 7:**
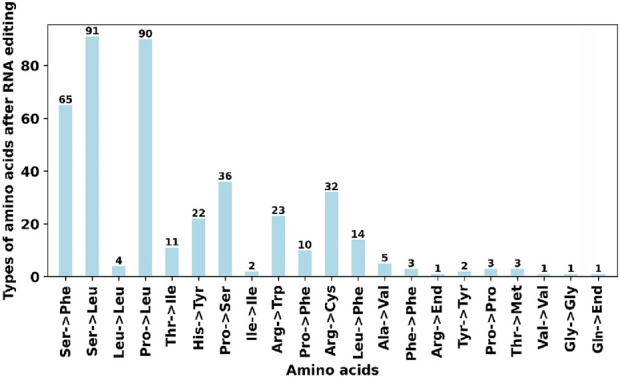
Types of amino acids after RNA editing.

RNA editing events were identified for the 31 unique PCGs from the *F. hirta* mitochondrial genome (Figure 6). The cutoff value was set to 0.9. Under this criterion, a total of 419 potential RNA editing sites were computationally identified across the 31 mitochondrial PCGs, all of which involved C-to-U base editing. Among the mitochondrial genes, the *nad4* gene had the highest number of RNA editing sites, with 47 identified edits. The second highest was the *nad7* gene, with 36 RNA editing events. According to the annotation results, there are a total of three RNA editing events related to translation, including the start codon ACG of nad1 and the stop codons CAA of atp9 and CGA of ccmFC, which are likely generated by RNA editing. Through C-to-U editing, these codons are converted into the start codon AUG and the stop codons UAA/UGA, respectively (Figure 7; Supplementary Table S7).

### 3.7 Synteny analysis of mitochondrial genome structure in *Ficus hirta*

**FIGURE 8:**
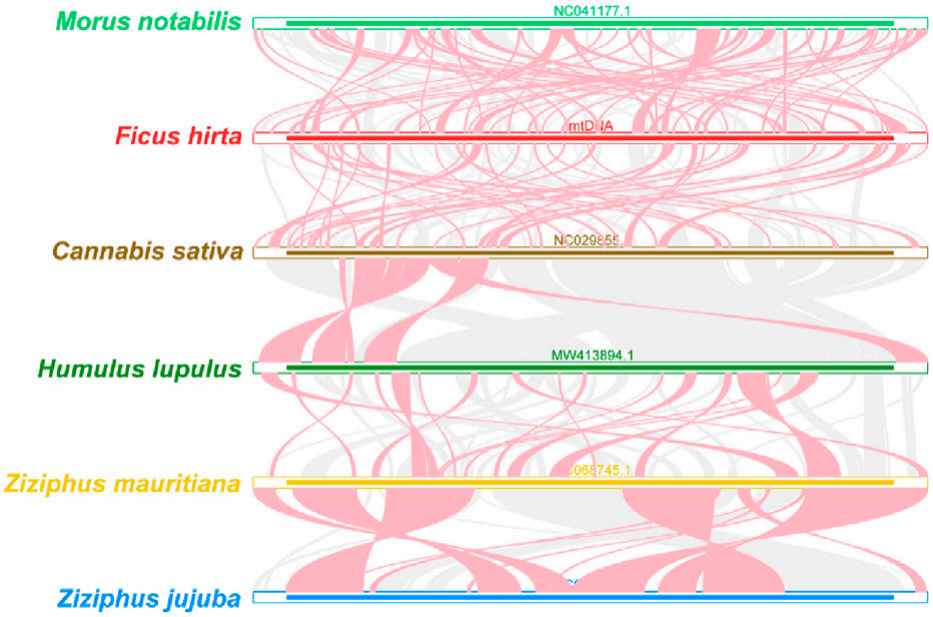
Synteny Analysis of Mitochondrial Genome Structure in *Ficus hirta*. The red arc areas represent regions of inversion, while the gray areas indicate regions with high homology.

As shown in Figure 8, the red arc areas represent regions of inversion, while the gray areas indicate regions with high homology. To better present the results, collinear blocks shorter than 0.5 kb were not retained in the analysis. Homologous collinear blocks were detected between *F. hirta* species and closely related species within the Rosales order, but these blocks were relatively short. Additionally, some blank regions were identified, representing sequences unique to the *F. hirta* species with no homology to other species. The results indicate that the arrangement of collinear blocks among the mitochondrial genomes of these six species is inconsistent, and the mitochondrial genome of *F. hirta* species has undergone extensive genome rearrangement compared to closely related species. The mitochondrial genome sequences of the six Rosales species are highly non-conserved in their arrangement, having experienced extremely frequent genome recombination.

## 4 Discussion

The assembly of the *F. hirta* mitochondrial genome provides valuable insights into its structure, gene content, and evolutionary conservation. The genome is organized as a circular molecule spanning 486,226 bp, with a GC content of 45.21%. The annotation identified 31 protein-coding genes, 19 tRNA genes, and three rRNA genes, all of which are highly conserved. Furthermore, annotation with *A. thaliana* and Liriodendron tulipifera confirms that *F. hirta* retains the core mitochondrial gene set, with no novel protein-coding genes identified.

One of the most intriguing findings is the presence of 15 homologous fragments shared between the mitochondrial and chloroplast genomes, suggesting significant interorganellar gene transfer events. These findings align with previous research in plants, where intracellular gene transfer between the chloroplast and mitochondrial genomes is a common phenomenon (Nguyen et al., 2020; Gui et al., 2016). The transfer of tRNA genes between organelles could be an adaptive mechanism that enhances genome plasticity. The study by Cui et al. (2021) supports the notion that chloroplast-to-mitochondrial transfers play a role in mitochondrial genome expansion. In species such as watermelon and melon, chloroplast-derived sequences account for 7.6% and 2.73% of the total mitochondrial genome, respectively. These findings highlight that mitochondria have a greater capacity to integrate foreign sequences, likely due to their more flexible DNA recombination mechanisms. Our findings in *F. hirta* are consistent with this trend, where small chloroplast-derived fragments were identified within the mitochondrial genome (Cui et al., 2021; Gao et al., 2020), supporting the hypothesis that such transfers contribute to the structural evolution of mitochondria (Cui et al., 2021).

The comparative phylogenetic analysis confirmed the close evolutionary relationship between *F. hirta* and Morus notabilis. However, notable differences in mitochondrial genome organization were observed between *F. hirta* and other members of the Rosales order, indicating frequent genome rearrangements within the group. Such rearrangements could be driven by the large repeat sequences identified in the genome, which may mediate recombination events.

Our codon usage bias analysis highlighted a preference for A/T-ending codons at the third position, a pattern commonly observed in plant mitochondrial genomes (Bi et al., 2020; Shidhi et al., 2021). From an evolutionary perspective, this bias is likely shaped by a combination of mutation pressure and natural selection (Ji et al., 2024; Cao et al., 2023). In terms of gene expression regulation, biased codon usage may impact mRNA stability and translation efficiency (Hia et al., 2019).

RNA editing in plant mitochondria is predominantly C-to-U conversions, which play a critical role in restoring conserved amino acid sequences that are functionally essential for mitochondrial proteins. This process helps correct mutations at the transcript level, ensuring proper protein functionality without requiring extensive changes in the DNA sequence (Ichinose and Sugita, 2016). The identification of 419 RNA editing sites, primarily involving C-to-U conversions, further underscores the complexity of gene expression in plant mitochondria. Such extensive RNA editing, especially in essential genes like nad4 and nad7, may be crucial for ensuring proper protein function. Our findings are based on predictions using PREP-Mt (v0.9), which is designed to detect C-to-U conversions, the most common form of RNA editing in plant mitochondria. Future research could leverage RNA-seq data to experimentally validate these predictions and explore the functional consequences of RNA editing.

## 5 Conclusion

In conclusion, the complete sequencing and annotation of the *F. hirta* mitochondrial genome provide valuable genomic resources that enhance our understanding of its biology and evolution. The intergenomic transfers, frequent genome rearrangements, and RNA editing events observed in *F. hirta* contribute to a broader understanding of plant mitochondrial genome dynamics. Future studies should focus on functional analyses of the transferred genes and their roles in mitochondrial function and adaptation.

## Data Availability

The original contributions presented in the study are publicly available. This data can be found here: NCBI’s BankIt, under the following accession numbers: mitochondrial genome data 2944861, and the chloroplast genome 2945015.

## Supplementary material

SUPPLEMENTARY TABLE S1 Annotated genes list in the mitogenome of *Ficus hirta*.

SUPPLEMENTARY TABLE S2 RSCU of each amino acid in the mitochondrial genome of *Ficus hirta*.

SUPPLEMENTARY TABLE S3 Tandem repeat sequences in the mitochondrial genome of Ficus hirta.

SUPPLEMENTARY TABLE S4 SSRs in the mitochondrial genome of *Ficus hirta*.

SUPPLEMENTARY TABLE S5 Dispersed repeat sequences in the mitochondrial genome of *Ficus hirta*.

SUPPLEMENTARY TABLE S6 The homologous DNA fragment in the *Ficus hirta* mitochondrial genome.

SUPPLEMENTARY TABLE S7 RNA editing.
