# Supplementary material for: Complete mitochondrial genome of Ficus hirta and its comparative analysis
Source: Front Genet. 2025 Apr 23;16:1530105. doi: 10.3389/fgene.2025.1530105 (PMC12055533; doi:10.3389/fgene.2025.1530105)
Supplement: Supplementary file 1 [file Supplementaryfile1.docx]

Supplementary Material

Supplementary Table 1. Annotated genes list in the mitogenome of *Ficus hirta*

| Group of genes | Name of genes |
| --- | --- |
| ATP synthase | *atp1*, *atp4*, *atp6*, *atp8*, *atp9* |
| NADH dehydrogenase | *nad1*, *nad2*, *nad3*, *nad4*, *nad4L*, *nad5*, *nad6*, *nad7*, *nad9* |
| Cytochrome *b* | *cob* |
| Cytochrome *c* biogenesis | *ccmB*, *ccmC*, *ccmFC*, *ccmFN* |
| Cytochrome *c* oxidase | *cox1*, *cox2*, *cox3* |
| Maturases | *matR* |
| Protein transport subunit | *mttB* |
| Ribosomal protein large subunit | *rpl16* |
| Ribosomal protein small subunit | *rps4*, *rps7*, *rps12*, *rps13* |
| Succinate dehydrogenase | *sdh3*, *sdh4* |
| Ribosome RNA | *rrn5*(*2), *rrn18*, *rrn26* |
| Transfer RNA | *trnA-UGC*(*2), *trnC-GCA*, *trnD-GUC*, *trnE-UUC*, *trnF-GAA*, *trnfM-CAU*, *trnI-CAU*, *trnI-GAU*, *trnK-UUU*, *trnL-CAA*, *trnM-CAU*, *trnN-GUU*, *trnP-UGG*(*2), *trnQ-UUG*, *trnR-ACG*, *trnS-GCU*, *trnV-GAC*, *trnW-CCA*(*2), *trnY-GUA* |

Copy numbers for specific genes are indicated in parentheses, such as rRNA (*2) for two copies.

Supplementary Table 2. RSCU of each amino acid in the mitochondrial genome of *Ficus hirta*

| Amino | Codon 1 | Codon 2 | Codon 3 | Codon 4 | Codon 5 | Codon 6 |
| --- | --- | --- | --- | --- | --- | --- |
|  | RSCU | RSCU | RSCU | RSCU | RSCU | RSCU |
| Ala | GCU | GCA | GCC | GCG |  |  |
|  | 1.63 | 0.95 | 0.94 | 0.48 |  |  |
| Arg | AGA | CGU | CGA | AGG | CGG | CGC |
|  | 1.37 | 1.32 | 1.25 | 0.73 | 0.7 | 0.63 |
| Asn | AAU | AAC |  |  |  |  |
|  | 1.37 | 0.63 |  |  |  |  |
| Asp | GAU | GAC |  |  |  |  |
|  | 1.37 | 0.63 |  |  |  |  |
| Cys | UGU | UGC |  |  |  |  |
|  | 1.26 | 0.74 |  |  |  |  |
| End | UAA | UGA | UAG |  |  |  |
|  | 1.55 | 1.14 | 0.31 |  |  |  |
| Gln | CAA | CAG |  |  |  |  |
|  | 1.54 | 0.46 |  |  |  |  |
| Glu | GAA | GAG |  |  |  |  |
|  | 1.42 | 0.58 |  |  |  |  |
| Gly | GGA | GGU | GGG | GGC |  |  |
|  | 1.45 | 1.35 | 0.68 | 0.52 |  |  |
| His | CAU | CAC |  |  |  |  |
|  | 1.48 | 0.52 |  |  |  |  |
| Ile | AUU | AUC | AUA |  |  |  |
|  | 1.39 | 0.81 | 0.8 |  |  |  |
| Leu | UUA | CUU | UUG | CUA | CUG | CUC |
|  | 1.48 | 1.27 | 1.15 | 1.02 | 0.55 | 0.54 |
| Lys | AAA | AAG |  |  |  |  |
|  | 1.25 | 0.75 |  |  |  |  |
| Met | AUG |  |  |  |  |  |
|  | 1 |  |  |  |  |  |
| Phe | UUU | UUC |  |  |  |  |
|  | 1.15 | 0.85 |  |  |  |  |
| Pro | CCU | CCA | CCC | CCG |  |  |
|  | 1.58 | 1.04 | 0.84 | 0.53 |  |  |
| Ser | UCU | UCA | AGU | UCC | UCG | AGC |
|  | 1.34 | 1.21 | 1.06 | 0.99 | 0.77 | 0.63 |
| Thr | ACU | ACC | ACA | ACG |  |  |
|  | 1.47 | 1.01 | 0.97 | 0.54 |  |  |
| Trp | UGG |  |  |  |  |  |
|  | 1 |  |  |  |  |  |
| Tyr | UAU | UAC |  |  |  |  |
|  | 1.54 | 0.46 |  |  |  |  |
| Val | GUU | GUA | GUG | GUC |  |  |
|  | 1.27 | 1.14 | 0.88 | 0.71 |  |  |

Supplementary Table 3 Tandem repeat sequences in the mitochondrial genome of *Ficus hirta*

| Indices | Period | Copy | Consensus | Percent | Percent | Score | A | C | G | T | Entropy |
| --- | --- | --- | --- | --- | --- | --- | --- | --- | --- | --- | --- |
|  | Size | Number | Size | Matches | Indels |  |  |  |  |  | (0-2) |
| 28795--28830 | 18 | 1.9 | 19 | 88 | 5 | 56 | 30 | 27 | 27 | 13 | 1.94 |
| 90150--90201 | 26 | 2 | 26 | 96 | 0 | 95 | 30 | 25 | 15 | 28 | 1.96 |
| 110764--110847 | 38 | 2.2 | 39 | 86 | 4 | 116 | 32 | 21 | 7 | 39 | 1.8 |
| 150952--150981 | 15 | 2 | 15 | 93 | 0 | 51 | 50 | 0 | 0 | 50 | 1 |
| 156010--156052 | 21 | 2 | 21 | 90 | 0 | 68 | 16 | 23 | 20 | 39 | 1.92 |
| 161840--161918 | 39 | 2 | 39 | 95 | 0 | 140 | 30 | 20 | 27 | 21 | 1.98 |
| 162818--162847 | 15 | 2 | 15 | 93 | 0 | 51 | 30 | 0 | 33 | 36 | 1.58 |
| 169421--169458 | 19 | 2 | 19 | 94 | 0 | 67 | 31 | 18 | 26 | 23 | 1.97 |
| 185328--185376 | 24 | 2 | 24 | 84 | 0 | 62 | 28 | 40 | 14 | 16 | 1.87 |
| 264558--264657 | 48 | 2.1 | 48 | 92 | 0 | 164 | 22 | 35 | 23 | 20 | 1.96 |
| 366395--366441 | 18 | 2.6 | 18 | 93 | 0 | 76 | 27 | 12 | 8 | 51 | 1.69 |
| 366411--366450 | 18 | 2.2 | 18 | 86 | 0 | 53 | 27 | 15 | 7 | 50 | 1.7 |
| 436492--436534 | 18 | 2.4 | 18 | 81 | 18 | 54 | 34 | 25 | 20 | 18 | 1.96 |
| 477577--477606 | 15 | 2 | 15 | 93 | 0 | 51 | 23 | 20 | 10 | 46 | 1.8 |
| 483276--483313 | 19 | 2 | 19 | 100 | 0 | 76 | 31 | 36 | 5 | 26 | 1.79 |

Supplementary Table 4 SSRs in the mitochondrial genome of *Ficus hirta*

| Mitochondrial genome | SSR nr. | SSR type | SSR | size | start | end |
| --- | --- | --- | --- | --- | --- | --- |
| *Ficus hirta* | 1 | p1 | (A)10 | 10 | 3134 | 3143 |
| *Ficus hirta* | 2 | p4 | (CTTG)3 | 12 | 21241 | 21252 |
| *Ficus hirta* | 3 | p2 | (TC)5 | 10 | 23389 | 23398 |
| *Ficus hirta* | 4 | p1 | (A)14 | 14 | 26399 | 26412 |
| *Ficus hirta* | 5 | p1 | (A)11 | 11 | 26608 | 26618 |
| *Ficus hirta* | 6 | p4 | (CTTA)3 | 12 | 29057 | 29068 |
| *Ficus hirta* | 7 | p2 | (TA)5 | 10 | 30896 | 30905 |
| *Ficus hirta* | 8 | p1 | (A)13 | 13 | 32466 | 32478 |
| *Ficus hirta* | 9 | p1 | (A)10 | 10 | 35610 | 35619 |
| *Ficus hirta* | 10 | p2 | (TC)5 | 10 | 36587 | 36596 |
| *Ficus hirta* | 11 | p2 | (TA)5 | 10 | 36597 | 36606 |
| *Ficus hirta* | 12 | p2 | (AG)5 | 10 | 36615 | 36624 |
| *Ficus hirta* | 13 | p4 | (GGAA)3 | 12 | 38306 | 38317 |
| *Ficus hirta* | 14 | p5 | (AAAAC)3 | 15 | 47296 | 47310 |
| *Ficus hirta* | 15 | p4 | (AGTT)3 | 12 | 51071 | 51082 |
| *Ficus hirta* | 16 | p4 | (GATA)3 | 12 | 58515 | 58526 |
| *Ficus hirta* | 17 | p4 | (AAAG)3 | 12 | 59150 | 59161 |
| *Ficus hirta* | 18 | p4 | (TTTC)3 | 12 | 63661 | 63672 |
| *Ficus hirta* | 19 | p1 | (A)10 | 10 | 68822 | 68831 |
| *Ficus hirta* | 20 | p3 | (TAA)4 | 12 | 71660 | 71671 |
| *Ficus hirta* | 21 | p1 | (A)10 | 10 | 72258 | 72267 |
| *Ficus hirta* | 22 | p1 | (A)11 | 11 | 74751 | 74761 |
| *Ficus hirta* | 23 | p1 | (A)10 | 10 | 81817 | 81826 |
| *Ficus hirta* | 24 | p1 | (T)11 | 11 | 82203 | 82213 |
| *Ficus hirta* | 25 | p1 | (A)10 | 10 | 84156 | 84165 |
| *Ficus hirta* | 26 | p1 | (A)10 | 10 | 88571 | 88580 |
| *Ficus hirta* | 27 | p3 | (TCC)4 | 12 | 89043 | 89054 |
| *Ficus hirta* | 28 | p1 | (A)11 | 11 | 89501 | 89511 |
| *Ficus hirta* | 29 | p4 | (TTCT)3 | 12 | 90502 | 90513 |
| *Ficus hirta* | 30 | p3 | (AAT)4 | 12 | 91911 | 91922 |
| *Ficus hirta* | 31 | p1 | (A)10 | 10 | 100672 | 100681 |
| *Ficus hirta* | 32 | p3 | (TTC)4 | 12 | 100909 | 100920 |
| *Ficus hirta* | 33 | p4 | (ATAC)3 | 12 | 101385 | 101396 |
| *Ficus hirta* | 34 | p4 | (TTTC)3 | 12 | 101398 | 101409 |
| *Ficus hirta* | 35 | p1 | (T)11 | 11 | 102127 | 102137 |
| *Ficus hirta* | 36 | p4 | (AATG)3 | 12 | 103562 | 103573 |
| *Ficus hirta* | 37 | p2 | (GA)6 | 12 | 104683 | 104694 |
| *Ficus hirta* | 38 | p2 | (AG)6 | 12 | 108552 | 108563 |
| *Ficus hirta* | 39 | p5 | (GGCAA)3 | 15 | 110092 | 110106 |
| *Ficus hirta* | 40 | p1 | (T)11 | 11 | 111342 | 111352 |
| *Ficus hirta* | 41 | p2 | (AT)6 | 12 | 111384 | 111395 |
| *Ficus hirta* | 42 | p4 | (AAGA)3 | 12 | 112004 | 112015 |
| *Ficus hirta* | 43 | p4 | (CTTA)3 | 12 | 113068 | 113079 |
| *Ficus hirta* | 44 | p4 | (AATT)3 | 12 | 114716 | 114727 |
| *Ficus hirta* | 45 | p1 | (T)10 | 10 | 123040 | 123049 |
| *Ficus hirta* | 46 | p1 | (T)12 | 12 | 128044 | 128055 |
| *Ficus hirta* | 47 | p4 | (ACCG)3 | 12 | 140110 | 140121 |
| *Ficus hirta* | 48 | p2 | (AT)8 | 16 | 150956 | 150971 |
| *Ficus hirta* | 49 | p2 | (TA)5 | 10 | 150972 | 150981 |
| *Ficus hirta* | 50 | p4 | (AAAG)3 | 12 | 151681 | 151692 |
| *Ficus hirta* | 51 | p2 | (AG)5 | 10 | 154255 | 154264 |
| *Ficus hirta* | 52 | p4 | (CCTA)3 | 12 | 154429 | 154440 |
| *Ficus hirta* | 53 | p1 | (A)12 | 12 | 161060 | 161071 |
| *Ficus hirta* | 54 | p2 | (TA)6 | 12 | 162575 | 162586 |
| *Ficus hirta* | 55 | p3 | (TAT)4 | 12 | 162607 | 162618 |
| *Ficus hirta* | 56 | p1 | (G)10 | 10 | 163306 | 163315 |
| *Ficus hirta* | 57 | p4 | (GAAT)3 | 12 | 166826 | 166837 |
| *Ficus hirta* | 58 | p4 | (AGCA)3 | 12 | 170883 | 170894 |
| *Ficus hirta* | 59 | p4 | (CTTT)3 | 12 | 174018 | 174029 |
| *Ficus hirta* | 60 | p1 | (T)11 | 11 | 175169 | 175179 |
| *Ficus hirta* | 61 | p2 | (TA)6 | 12 | 177260 | 177271 |
| *Ficus hirta* | 62 | p4 | (TTCT)3 | 12 | 177319 | 177330 |
| *Ficus hirta* | 63 | p1 | (T)11 | 11 | 177413 | 177423 |
| *Ficus hirta* | 64 | p4 | (CTTA)3 | 12 | 177761 | 177772 |
| *Ficus hirta* | 65 | p2 | (TA)5 | 10 | 181994 | 182003 |
| *Ficus hirta* | 66 | p1 | (A)10 | 10 | 191844 | 191853 |
| *Ficus hirta* | 67 | p2 | (TA)5 | 10 | 193543 | 193552 |
| *Ficus hirta* | 68 | p2 | (GA)5 | 10 | 193860 | 193869 |
| *Ficus hirta* | 69 | p1 | (A)10 | 10 | 197514 | 197523 |
| *Ficus hirta* | 70 | p2 | (TC)5 | 10 | 199033 | 199042 |
| *Ficus hirta* | 71 | p4 | (AAAG)3 | 12 | 199115 | 199126 |
| *Ficus hirta* | 72 | p1 | (T)10 | 10 | 201069 | 201078 |
| *Ficus hirta* | 73 | p2 | (AG)5 | 10 | 205409 | 205418 |
| *Ficus hirta* | 74 | p1 | (T)10 | 10 | 213200 | 213209 |
| *Ficus hirta* | 75 | p2 | (TA)6 | 12 | 218518 | 218529 |
| *Ficus hirta* | 76 | p4 | (TTTG)3 | 12 | 223400 | 223411 |
| *Ficus hirta* | 77 | p3 | (GTA)4 | 12 | 227890 | 227901 |
| *Ficus hirta* | 78 | p2 | (AT)5 | 10 | 229149 | 229158 |
| *Ficus hirta* | 79 | p2 | (CT)5 | 10 | 231486 | 231495 |
| *Ficus hirta* | 80 | p1 | (A)14 | 14 | 237302 | 237315 |
| *Ficus hirta* | 81 | p4 | (TTAT)3 | 12 | 238098 | 238109 |
| *Ficus hirta* | 82 | p3 | (ACT)4 | 12 | 240313 | 240324 |
| *Ficus hirta* | 83 | p2 | (GA)5 | 10 | 241519 | 241528 |
| *Ficus hirta* | 84 | p4 | (TTCA)3 | 12 | 246903 | 246914 |
| *Ficus hirta* | 85 | p2 | (AG)5 | 10 | 254704 | 254713 |
| *Ficus hirta* | 86 | p4 | (TTAA)3 | 12 | 255304 | 255315 |
| *Ficus hirta* | 87 | p5 | (TAAAT)4 | 20 | 256336 | 256355 |
| *Ficus hirta* | 88 | p4 | (AGAA)3 | 12 | 262003 | 262014 |
| *Ficus hirta* | 89 | p1 | (A)10 | 10 | 263857 | 263866 |
| *Ficus hirta* | 90 | p1 | (T)10 | 10 | 264135 | 264144 |
| *Ficus hirta* | 91 | p1 | (A)11 | 11 | 269613 | 269623 |
| *Ficus hirta* | 92 | p3 | (TTA)4 | 12 | 271744 | 271755 |
| *Ficus hirta* | 93 | p1 | (T)12 | 12 | 272537 | 272548 |
| *Ficus hirta* | 94 | p4 | (GACC)3 | 12 | 273949 | 273960 |
| *Ficus hirta* | 95 | p1 | (T)10 | 10 | 277740 | 277749 |
| *Ficus hirta* | 96 | p2 | (TA)6 | 12 | 279331 | 279342 |
| *Ficus hirta* | 97 | p2 | (AG)5 | 10 | 284671 | 284680 |
| *Ficus hirta* | 98 | p1 | (T)12 | 12 | 285739 | 285750 |
| *Ficus hirta* | 99 | p4 | (TGAC)3 | 12 | 288367 | 288378 |
| *Ficus hirta* | 100 | p2 | (GA)6 | 12 | 295266 | 295277 |
| *Ficus hirta* | 101 | p1 | (A)12 | 12 | 296999 | 297010 |
| *Ficus hirta* | 102 | p3 | (TTG)4 | 12 | 298397 | 298408 |
| *Ficus hirta* | 103 | p1 | (A)11 | 11 | 300280 | 300290 |
| *Ficus hirta* | 104 | p1 | (T)12 | 12 | 300302 | 300313 |
| *Ficus hirta* | 105 | p1 | (T)11 | 11 | 300874 | 300884 |
| *Ficus hirta* | 106 | p2 | (AG)5 | 10 | 301041 | 301050 |
| *Ficus hirta* | 107 | p1 | (T)10 | 10 | 302257 | 302266 |
| *Ficus hirta* | 108 | p4 | (GTTG)3 | 12 | 303999 | 304010 |
| *Ficus hirta* | 109 | p2 | (AT)5 | 10 | 309083 | 309092 |
| *Ficus hirta* | 110 | p3 | (TCC)4 | 12 | 314644 | 314655 |
| *Ficus hirta* | 111 | p4 | (AAAG)3 | 12 | 320992 | 321003 |
| *Ficus hirta* | 112 | p1 | (T)10 | 10 | 328854 | 328863 |
| *Ficus hirta* | 113 | p1 | (T)10 | 10 | 329976 | 329985 |
| *Ficus hirta* | 114 | p3 | (TAT)4 | 12 | 334062 | 334073 |
| *Ficus hirta* | 115 | p4 | (GTTT)3 | 12 | 334222 | 334233 |
| *Ficus hirta* | 116 | p1 | (A)10 | 10 | 335798 | 335807 |
| *Ficus hirta* | 117 | p3 | (GAA)4 | 12 | 341824 | 341835 |
| *Ficus hirta* | 118 | p4 | (CAAT)3 | 12 | 345075 | 345086 |
| *Ficus hirta* | 119 | p4 | (TTTA)3 | 12 | 350382 | 350393 |
| *Ficus hirta* | 120 | p4 | (GTGA)3 | 12 | 353660 | 353671 |
| *Ficus hirta* | 121 | p2 | (AT)5 | 10 | 360897 | 360906 |
| *Ficus hirta* | 122 | p2 | (TA)5 | 10 | 360934 | 360943 |
| *Ficus hirta* | 123 | p3 | (AAG)4 | 12 | 364036 | 364047 |
| *Ficus hirta* | 124 | p1 | (A)10 | 10 | 371715 | 371724 |
| *Ficus hirta* | 125 | p2 | (TC)6 | 12 | 387142 | 387153 |
| *Ficus hirta* | 126 | p3 | (GTT)4 | 12 | 388839 | 388850 |
| *Ficus hirta* | 127 | p4 | (CTTT)3 | 12 | 391162 | 391173 |
| *Ficus hirta* | 128 | p1 | (T)12 | 12 | 395561 | 395572 |
| *Ficus hirta* | 129 | p4 | (TGAA)3 | 12 | 402916 | 402927 |
| *Ficus hirta* | 130 | p1 | (T)14 | 14 | 403957 | 403970 |
| *Ficus hirta* | 131 | p1 | (A)13 | 13 | 409556 | 409568 |
| *Ficus hirta* | 132 | p4 | (TCGC)3 | 12 | 410536 | 410547 |
| *Ficus hirta* | 133 | p1 | (A)10 | 10 | 414179 | 414188 |
| *Ficus hirta* | 134 | p1 | (T)11 | 11 | 417936 | 417946 |
| *Ficus hirta* | 135 | p4 | (TCTA)3 | 12 | 425518 | 425529 |
| *Ficus hirta* | 136 | p1 | (A)10 | 10 | 428412 | 428421 |
| *Ficus hirta* | 137 | p1 | (T)12 | 12 | 429151 | 429162 |
| *Ficus hirta* | 138 | p3 | (AGA)4 | 12 | 429167 | 429178 |
| *Ficus hirta* | 139 | p1 | (C)10 | 10 | 434448 | 434457 |
| *Ficus hirta* | 140 | p4 | (GCCG)3 | 12 | 449751 | 449762 |
| *Ficus hirta* | 141 | p2 | (TA)5 | 10 | 454370 | 454379 |
| *Ficus hirta* | 142 | p1 | (A)12 | 12 | 462533 | 462544 |
| *Ficus hirta* | 143 | p1 | (A)11 | 11 | 462968 | 462978 |
| *Ficus hirta* | 144 | p4 | (ATCT)3 | 12 | 463844 | 463855 |
| *Ficus hirta* | 145 | p1 | (A)16 | 16 | 463990 | 464005 |
| *Ficus hirta* | 146 | p1 | (T)12 | 12 | 464595 | 464606 |
| *Ficus hirta* | 147 | p2 | (CT)5 | 10 | 465699 | 465708 |
| *Ficus hirta* | 148 | p3 | (GAT)4 | 12 | 465979 | 465990 |
| *Ficus hirta* | 149 | p1 | (C)10 | 10 | 468417 | 468426 |
| *Ficus hirta* | 150 | p2 | (CT)5 | 10 | 469541 | 469550 |
| *Ficus hirta* | 151 | p3 | (TTC)4 | 12 | 474284 | 474295 |
| *Ficus hirta* | 152 | p1 | (A)12 | 12 | 484880 | 484891 |
| *Ficus hirta* | 153 | p4 | (TTAT)3 | 12 | 485854 | 485865 |

Supplementary Table 5 Dispersed repeat sequences in the mitochondrial genome of *Ficus hirta*

| The repeat length of the first part | The starting site of the first part | Matching direction | The repeat length of the second part | The starting site of the second part | Interval distance of repeats | E-value |
| --- | --- | --- | --- | --- | --- | --- |
| 12060 | 205640 | P | 12060 | 367223 | 0 | 0.00E+00 |
| 521 | 237949 | F | 521 | 485705 | 0 | 0.00E+00 |
| 368 | 105015 | P | 368 | 433316 | 0 | 1.84E-211 |
| 342 | 81280 | F | 342 | 275769 | 0 | 8.28E-196 |
| 270 | 408160 | P | 270 | 429245 | 0 | 1.85E-152 |
| 230 | 75175 | P | 230 | 130464 | 0 | 2.23E-128 |
| 198 | 409855 | F | 198 | 433672 | -1 | 2.45E-106 |
| 154 | 406435 | F | 154 | 446689 | 0 | 1.28E-82 |
| 128 | 173523 | P | 128 | 396666 | -1 | 2.21E-64 |
| 97 | 409956 | F | 97 | 433773 | 0 | 2.65E-48 |
| 103 | 110564 | P | 103 | 291796 | -2 | 3.06E-47 |
| 88 | 100723 | P | 88 | 315538 | 0 | 6.94E-43 |
| 88 | 247139 | F | 88 | 296813 | -3 | 2.06E-36 |
| 88 | 281530 | P | 88 | 331556 | -3 | 2.06E-36 |
| 77 | 133085 | F | 77 | 426386 | 0 | 2.91E-36 |
| 87 | 134586 | F | 87 | 183356 | -3 | 7.95E-36 |
| 76 | 421772 | F | 76 | 455784 | 0 | 1.16E-35 |
| 72 | 131155 | F | 72 | 406392 | 0 | 2.98E-33 |
| 70 | 281548 | P | 70 | 331556 | 0 | 4.77E-32 |
| 79 | 200083 | F | 79 | 405566 | -3 | 3.89E-31 |
| 72 | 237223 | P | 72 | 245600 | -2 | 6.86E-29 |
| 67 | 200095 | F | 67 | 405578 | -1 | 6.14E-28 |
| 62 | 237233 | P | 62 | 245600 | 0 | 3.13E-27 |
| 61 | 182099 | F | 61 | 296795 | 0 | 1.25E-26 |
| 64 | 247163 | F | 64 | 296837 | -1 | 3.75E-26 |
| 60 | 100605 | F | 60 | 315621 | 0 | 5.00E-26 |
| 63 | 77138 | F | 63 | 132572 | -1 | 1.48E-25 |
| 58 | 93179 | F | 58 | 137316 | 0 | 8.00E-25 |
| 68 | 112024 | F | 68 | 245627 | -3 | 1.03E-24 |
| 56 | 99111 | P | 56 | 384932 | 0 | 1.28E-23 |
| 65 | 135164 | F | 65 | 183930 | -3 | 5.76E-23 |
| 60 | 112032 | F | 60 | 245635 | -2 | 7.97E-22 |
| 60 | 213142 | F | 60 | 317440 | -2 | 7.97E-22 |
| 60 | 317440 | P | 60 | 371721 | -2 | 7.97E-22 |
| 54 | 110613 | P | 54 | 291796 | -1 | 3.32E-20 |
| 50 | 278714 | P | 50 | 410032 | 0 | 5.25E-20 |
| 56 | 34966 | P | 56 | 296857 | -2 | 1.77E-19 |
| 49 | 34973 | F | 49 | 182972 | 0 | 2.10E-19 |
| 49 | 45105 | P | 49 | 296342 | 0 | 2.10E-19 |
| 49 | 164731 | F | 49 | 181709 | 0 | 2.10E-19 |
| 52 | 103286 | F | 52 | 470905 | -1 | 5.11E-19 |
| 51 | 239681 | F | 51 | 302180 | -1 | 2.01E-18 |
| 47 | 77154 | F | 47 | 132588 | 0 | 3.36E-18 |
| 53 | 202197 | P | 53 | 263899 | -2 | 1.02E-17 |
| 46 | 35082 | F | 46 | 393114 | 0 | 1.34E-17 |
| 49 | 64811 | F | 49 | 201539 | -1 | 3.08E-17 |
| 49 | 182972 | P | 49 | 296857 | -1 | 3.08E-17 |
| 49 | 273521 | P | 49 | 343023 | -1 | 3.08E-17 |
| 55 | 331279 | P | 55 | 428569 | -3 | 3.63E-17 |
| 52 | 464339 | P | 52 | 464339 | -2 | 3.91E-17 |
| 54 | 112051 | F | 54 | 245654 | -3 | 1.37E-16 |
| 54 | 334339 | F | 54 | 485129 | -3 | 1.37E-16 |
| 51 | 120131 | F | 51 | 143330 | -2 | 1.50E-16 |
| 44 | 34978 | P | 44 | 247183 | 0 | 2.15E-16 |
| 44 | 112315 | F | 44 | 468966 | 0 | 2.15E-16 |
| 44 | 182977 | P | 44 | 247183 | 0 | 2.15E-16 |
| 44 | 315504 | F | 44 | 379626 | 0 | 2.15E-16 |
| 44 | 434591 | F | 44 | 435153 | 0 | 2.15E-16 |
| 42 | 126222 | F | 42 | 421730 | 0 | 3.44E-15 |
| 48 | 135181 | F | 48 | 183947 | -2 | 8.52E-15 |
| 50 | 73873 | P | 50 | 233168 | -3 | 2.78E-14 |
| 40 | 241704 | P | 40 | 345154 | 0 | 5.50E-14 |
| 49 | 120115 | F | 49 | 143314 | -3 | 1.04E-13 |
| 49 | 144800 | P | 49 | 456997 | -3 | 1.04E-13 |
| 49 | 436102 | F | 49 | 460132 | -3 | 1.04E-13 |
| 43 | 202207 | P | 43 | 263899 | -1 | 1.11E-13 |
| 39 | 88703 | P | 39 | 258773 | 0 | 2.20E-13 |
| 39 | 236998 | F | 39 | 403772 | 0 | 2.20E-13 |
| 39 | 283235 | P | 39 | 449397 | 0 | 2.20E-13 |
| 48 | 104336 | P | 48 | 308547 | -3 | 3.92E-13 |
| 48 | 118548 | P | 48 | 179790 | -3 | 3.92E-13 |
| 45 | 112060 | F | 45 | 245663 | -2 | 4.79E-13 |
| 45 | 436318 | F | 45 | 460350 | -2 | 4.79E-13 |
| 38 | 289633 | P | 38 | 296055 | 0 | 8.80E-13 |
| 47 | 207630 | F | 47 | 208365 | -3 | 1.47E-12 |
| 47 | 207630 | P | 47 | 376511 | -3 | 1.47E-12 |
| 47 | 208365 | P | 47 | 377246 | -3 | 1.47E-12 |
| 47 | 264557 | F | 47 | 264605 | -3 | 1.47E-12 |
| 47 | 376511 | F | 47 | 377246 | -3 | 1.47E-12 |
| 37 | 230107 | F | 37 | 286618 | 0 | 3.52E-12 |
| 43 | 55160 | F | 43 | 64912 | -2 | 6.98E-12 |
| 43 | 182117 | F | 43 | 247139 | -2 | 6.98E-12 |
| 36 | 131223 | F | 36 | 461720 | 0 | 1.41E-11 |
| 36 | 175436 | F | 36 | 229428 | 0 | 1.41E-11 |
| 36 | 309201 | P | 36 | 446864 | 0 | 1.41E-11 |
| 36 | 436836 | F | 36 | 464562 | 0 | 1.41E-11 |
| 45 | 112024 | P | 45 | 237223 | -3 | 2.06E-11 |
| 45 | 331295 | P | 45 | 428563 | -3 | 2.06E-11 |
| 45 | 435454 | F | 45 | 459468 | -3 | 2.06E-11 |
| 39 | 74425 | P | 39 | 232630 | -1 | 2.57E-11 |
| 42 | 201481 | P | 42 | 309336 | -2 | 2.66E-11 |
| 35 | 112755 | P | 35 | 273371 | 0 | 5.63E-11 |
| 35 | 119229 | F | 35 | 435538 | 0 | 5.63E-11 |
| 35 | 182062 | F | 35 | 453200 | 0 | 5.63E-11 |
| 38 | 184231 | F | 38 | 484456 | -1 | 1.00E-10 |
| 41 | 1735 | F | 41 | 97541 | -2 | 1.01E-10 |
| 41 | 113881 | P | 41 | 143343 | -2 | 1.01E-10 |
| 34 | 19139 | F | 34 | 73295 | 0 | 2.25E-10 |
| 34 | 94680 | P | 34 | 247416 | 0 | 2.25E-10 |
| 34 | 182160 | F | 34 | 247179 | 0 | 2.25E-10 |
| 34 | 182160 | F | 34 | 296853 | 0 | 2.25E-10 |
| 34 | 417366 | P | 34 | 430810 | 0 | 2.25E-10 |
| 43 | 73861 | P | 43 | 233187 | -3 | 2.86E-10 |
| 43 | 91114 | P | 43 | 186784 | -3 | 2.86E-10 |
| 43 | 118229 | P | 43 | 180123 | -3 | 2.86E-10 |
| 43 | 118604 | P | 43 | 143343 | -3 | 2.86E-10 |
| 43 | 207373 | F | 43 | 383304 | -3 | 2.86E-10 |
| 43 | 377507 | P | 43 | 383304 | -3 | 2.86E-10 |
| 40 | 161839 | F | 40 | 161878 | -2 | 3.86E-10 |
| 40 | 331304 | P | 40 | 428559 | -2 | 3.86E-10 |
| 33 | 244699 | F | 33 | 391439 | 0 | 9.01E-10 |
| 33 | 255944 | P | 33 | 436835 | 0 | 9.01E-10 |
| 42 | 113881 | F | 42 | 118606 | -3 | 1.07E-09 |
| 42 | 201429 | P | 42 | 309370 | -3 | 1.07E-09 |
| 42 | 334470 | F | 42 | 485268 | -3 | 1.07E-09 |
| 39 | 213088 | F | 39 | 317390 | -2 | 1.47E-09 |
| 39 | 317390 | P | 39 | 371796 | -2 | 1.47E-09 |
| 36 | 182249 | P | 36 | 391389 | -1 | 1.52E-09 |
| 36 | 197324 | P | 36 | 348175 | -1 | 1.52E-09 |
| 32 | 84618 | P | 32 | 151535 | 0 | 3.6E-09 |
| 32 | 200130 | F | 32 | 405613 | 0 | 3.6E-09 |
| 32 | 222834 | P | 32 | 335827 | 0 | 3.6E-09 |
| 32 | 255944 | P | 32 | 464562 | 0 | 3.6E-09 |
| 32 | 273538 | P | 32 | 343023 | 0 | 3.6E-09 |
| 41 | 55233 | F | 41 | 64999 | -3 | 3.96E-09 |
| 41 | 118615 | P | 41 | 143334 | -3 | 3.96E-09 |
| 41 | 128043 | P | 41 | 237272 | -3 | 3.96E-09 |
| 41 | 180859 | P | 41 | 382761 | -3 | 3.96E-09 |
| 35 | 12356 | P | 35 | 135252 | -1 | 5.91E-09 |
| 35 | 393875 | F | 35 | 409303 | -1 | 5.91E-09 |
| 35 | 484390 | P | 35 | 484390 | -1 | 5.91E-09 |
| 31 | 34991 | P | 31 | 296857 | 0 | 1.44E-08 |
| 31 | 48005 | P | 31 | 92399 | 0 | 1.44E-08 |
| 31 | 182990 | P | 31 | 296857 | 0 | 1.44E-08 |
| 40 | 104939 | F | 40 | 420228 | -3 | 1.47E-08 |
| 40 | 105267 | F | 40 | 468974 | -3 | 1.47E-08 |
| 40 | 120614 | F | 40 | 403135 | -3 | 1.47E-08 |
| 40 | 334484 | F | 40 | 485282 | -3 | 1.47E-08 |
| 40 | 408111 | P | 40 | 429528 | -3 | 1.47E-08 |
| 40 | 433392 | P | 40 | 468974 | -3 | 1.47E-08 |
| 37 | 112032 | P | 37 | 237223 | -2 | 2.11E-08 |
| 37 | 113866 | F | 37 | 118591 | -2 | 2.11E-08 |
| 37 | 296718 | P | 37 | 393106 | -2 | 2.11E-08 |
| 34 | 173435 | F | 34 | 417847 | -1 | 0.000000023 |
| 34 | 199979 | P | 34 | 264335 | -1 | 0.000000023 |
| 34 | 253816 | P | 34 | 289202 | -1 | 0.000000023 |
| 39 | 19974 | P | 39 | 443264 | -3 | 5.43E-08 |
| 39 | 54910 | F | 39 | 93721 | -3 | 5.43E-08 |
| 39 | 73310 | P | 39 | 233722 | -3 | 5.43E-08 |
| 39 | 74070 | P | 39 | 232990 | -3 | 5.43E-08 |
| 39 | 78495 | F | 39 | 350200 | -3 | 5.43E-08 |
| 39 | 104349 | P | 39 | 308543 | -3 | 5.43E-08 |
| 39 | 120131 | F | 39 | 179726 | -3 | 5.43E-08 |
| 39 | 143330 | F | 39 | 179726 | -3 | 5.43E-08 |
| 39 | 161640 | F | 39 | 316886 | -3 | 5.43E-08 |
| 39 | 177757 | F | 39 | 244388 | -3 | 5.43E-08 |
| 39 | 180461 | P | 39 | 207246 | -3 | 5.43E-08 |
| 39 | 180461 | F | 39 | 377638 | -3 | 5.43E-08 |
| 39 | 180944 | F | 39 | 309502 | -3 | 5.43E-08 |
| 39 | 264570 | F | 39 | 264618 | -3 | 5.43E-08 |
| 30 | 34992 | P | 30 | 182164 | 0 | 5.77E-08 |
| 30 | 109645 | F | 30 | 398429 | 0 | 5.77E-08 |
| 30 | 128148 | F | 30 | 438867 | 0 | 5.77E-08 |
| 30 | 182164 | P | 30 | 182991 | 0 | 5.77E-08 |
| 36 | 36732 | P | 36 | 278725 | -2 | 7.98E-08 |
| 36 | 36732 | F | 36 | 410035 | -2 | 7.98E-08 |
| 36 | 257733 | F | 36 | 298278 | -2 | 7.98E-08 |
| 36 | 315483 | P | 36 | 435186 | -2 | 7.98E-08 |
| 33 | 78302 | P | 33 | 239110 | -1 | 8.92E-08 |
| 33 | 85359 | F | 33 | 479880 | -1 | 8.92E-08 |
| 33 | 112238 | F | 33 | 442621 | -1 | 8.92E-08 |
| 33 | 311663 | P | 33 | 472581 | -1 | 8.92E-08 |
| 38 | 19367 | F | 38 | 73537 | -3 | 0.0000002 |
| 38 | 105276 | F | 38 | 112332 | -3 | 0.0000002 |
| 38 | 112332 | P | 38 | 433385 | -3 | 0.0000002 |
| 38 | 118243 | P | 38 | 180114 | -3 | 0.0000002 |
| 38 | 232990 | F | 38 | 443372 | -3 | 0.0000002 |
| 35 | 81307 | F | 35 | 438780 | -2 | 0.000000302 |
| 35 | 118204 | P | 35 | 180156 | -2 | 0.000000302 |
| 35 | 134473 | F | 35 | 183244 | -2 | 0.000000302 |
| 35 | 180871 | F | 35 | 309429 | -2 | 0.000000302 |
| 35 | 275796 | F | 35 | 438780 | -2 | 0.000000302 |
| 35 | 377515 | P | 35 | 383304 | -2 | 0.000000302 |
| 32 | 1007 | F | 32 | 261425 | -1 | 0.000000346 |
| 32 | 51819 | F | 32 | 205683 | -1 | 0.000000346 |
| 32 | 51819 | P | 32 | 379208 | -1 | 0.000000346 |
| 32 | 113890 | P | 32 | 143343 | -1 | 0.000000346 |
| 32 | 134038 | F | 32 | 182403 | -1 | 0.000000346 |
| 32 | 330985 | P | 32 | 442561 | -1 | 0.000000346 |
| 37 | 180527 | P | 37 | 383099 | -3 | 0.000000738 |
| 37 | 208771 | F | 37 | 274454 | -3 | 0.000000738 |
| 37 | 274454 | P | 37 | 376115 | -3 | 0.000000738 |
| 37 | 436912 | P | 37 | 442345 | -3 | 0.000000738 |
| 37 | 439469 | P | 37 | 448131 | -3 | 0.000000738 |
| 34 | 3901 | F | 34 | 416751 | -2 | 0.00000114 |
| 34 | 19317 | F | 34 | 73487 | -2 | 0.00000114 |
| 34 | 39990 | F | 34 | 40032 | -2 | 0.00000114 |
| 31 | 31764 | F | 31 | 312523 | -1 | 0.00000134 |
| 31 | 64770 | F | 31 | 122825 | -1 | 0.00000134 |
| 31 | 131198 | F | 31 | 446689 | -1 | 0.00000134 |
| 31 | 181957 | P | 31 | 247120 | -1 | 0.00000134 |
| 31 | 194233 | P | 31 | 440511 | -1 | 0.00000134 |
| 31 | 230343 | F | 31 | 316681 | -1 | 0.00000134 |
| 31 | 245595 | F | 31 | 264215 | -1 | 0.00000134 |
| 36 | 52613 | P | 36 | 179132 | -3 | 0.00000271 |
| 36 | 74542 | P | 36 | 206963 | -3 | 0.00000271 |
| 36 | 74542 | F | 36 | 377924 | -3 | 0.00000271 |
| 36 | 91222 | F | 36 | 348726 | -3 | 0.00000271 |
| 36 | 91233 | F | 36 | 348737 | -3 | 0.00000271 |
| 36 | 104284 | P | 36 | 308611 | -3 | 0.00000271 |
| 36 | 134309 | F | 36 | 182719 | -3 | 0.00000271 |
| 36 | 152283 | F | 36 | 152342 | -3 | 0.00000271 |
| 33 | 20213 | P | 33 | 384832 | -2 | 0.00000428 |
| 33 | 105267 | F | 33 | 112323 | -2 | 0.00000428 |
| 33 | 112323 | P | 33 | 433399 | -2 | 0.00000428 |
| 30 | 2054 | P | 30 | 479829 | -1 | 0.00000519 |
| 30 | 19899 | P | 30 | 332404 | -1 | 0.00000519 |
| 30 | 81300 | P | 30 | 436848 | -1 | 0.00000519 |
| 30 | 134140 | F | 30 | 182552 | -1 | 0.00000519 |
| 30 | 135019 | F | 30 | 183783 | -1 | 0.00000519 |
| 30 | 201663 | F | 30 | 252623 | -1 | 0.00000519 |
| 30 | 233186 | F | 30 | 443571 | -1 | 0.00000519 |
| 30 | 275789 | P | 30 | 436848 | -1 | 0.00000519 |
| 30 | 437994 | F | 30 | 461597 | -1 | 0.00000519 |
| 30 | 438773 | P | 30 | 464574 | -1 | 0.00000519 |
| 35 | 19247 | F | 35 | 73417 | -3 | 0.00000995 |
| 35 | 19698 | F | 35 | 73870 | -3 | 0.00000995 |
| 35 | 19987 | F | 35 | 74164 | -3 | 0.00000995 |
| 35 | 52589 | P | 35 | 179157 | -3 | 0.00000995 |
| 35 | 54781 | F | 35 | 93601 | -3 | 0.00000995 |
| 35 | 73657 | P | 35 | 233399 | -3 | 0.00000995 |
| 35 | 74440 | P | 35 | 442984 | -3 | 0.00000995 |
| 35 | 78012 | F | 35 | 150788 | -3 | 0.00000995 |
| 35 | 233174 | F | 35 | 443559 | -3 | 0.00000995 |
| 35 | 235322 | F | 35 | 443562 | -3 | 0.00000995 |
| 35 | 309423 | P | 35 | 382761 | -3 | 0.00000995 |
| 35 | 334374 | F | 35 | 485164 | -3 | 0.00000995 |
| 35 | 435667 | F | 35 | 459681 | -3 | 0.00000995 |
| 32 | 26807 | P | 32 | 300875 | -2 | 0.0000161 |
| 32 | 74590 | P | 32 | 442837 | -2 | 0.0000161 |
| 32 | 98522 | P | 32 | 407564 | -2 | 0.0000161 |
| 32 | 113779 | P | 32 | 179850 | -2 | 0.0000161 |
| 32 | 118564 | P | 32 | 179790 | -2 | 0.0000161 |
| 32 | 144817 | P | 32 | 456997 | -2 | 0.0000161 |
| 32 | 145591 | P | 32 | 201966 | -2 | 0.0000161 |
| 32 | 180868 | P | 32 | 382761 | -2 | 0.0000161 |
| 32 | 181917 | F | 32 | 278721 | -2 | 0.0000161 |
| 32 | 181917 | P | 32 | 410043 | -2 | 0.0000161 |
| 32 | 206970 | F | 32 | 442888 | -2 | 0.0000161 |
| 32 | 278725 | F | 32 | 465718 | -2 | 0.0000161 |
| 32 | 285541 | F | 32 | 316127 | -2 | 0.0000161 |
| 32 | 377921 | P | 32 | 442888 | -2 | 0.0000161 |
| 32 | 410039 | P | 32 | 465718 | -2 | 0.0000161 |
| 34 | 134746 | F | 34 | 183512 | -3 | 0.0000364 |
| 34 | 134755 | F | 34 | 183521 | -3 | 0.0000364 |
| 34 | 172075 | P | 34 | 257558 | -3 | 0.0000364 |
| 34 | 233163 | F | 34 | 332582 | -3 | 0.0000364 |
| 31 | 2102 | F | 31 | 2148 | -2 | 0.0000603 |
| 31 | 52772 | F | 31 | 384651 | -2 | 0.0000603 |
| 31 | 52864 | P | 31 | 178889 | -2 | 0.0000603 |
| 31 | 81306 | P | 31 | 348003 | -2 | 0.0000603 |
| 31 | 91130 | P | 31 | 186780 | -2 | 0.0000603 |
| 31 | 93098 | P | 31 | 212403 | -2 | 0.0000603 |
| 31 | 93098 | F | 31 | 372489 | -2 | 0.0000603 |
| 31 | 105276 | F | 31 | 468983 | -2 | 0.0000603 |
| 31 | 113484 | F | 31 | 118219 | -2 | 0.0000603 |
| 31 | 122401 | F | 31 | 291127 | -2 | 0.0000603 |
| 31 | 180952 | F | 31 | 309510 | -2 | 0.0000603 |
| 31 | 207193 | F | 31 | 383105 | -2 | 0.0000603 |
| 31 | 213126 | P | 31 | 317417 | -2 | 0.0000603 |
| 31 | 244678 | P | 31 | 466840 | -2 | 0.0000603 |
| 31 | 249384 | P | 31 | 293493 | -2 | 0.0000603 |
| 31 | 264251 | P | 31 | 340115 | -2 | 0.0000603 |
| 31 | 275795 | P | 31 | 348003 | -2 | 0.0000603 |
| 31 | 317419 | F | 31 | 371768 | -2 | 0.0000603 |
| 31 | 334493 | F | 31 | 485291 | -2 | 0.0000603 |
| 31 | 355318 | F | 31 | 465868 | -2 | 0.0000603 |
| 31 | 377699 | P | 31 | 383105 | -2 | 0.0000603 |
| 33 | 12460 | P | 33 | 135151 | -3 | 0.000133 |
| 33 | 19670 | P | 33 | 235361 | -3 | 0.000133 |
| 33 | 36740 | P | 33 | 465713 | -3 | 0.000133 |
| 33 | 74080 | P | 33 | 443368 | -3 | 0.000133 |
| 33 | 99216 | P | 33 | 131140 | -3 | 0.000133 |
| 33 | 118252 | P | 33 | 180110 | -3 | 0.000133 |
| 33 | 233186 | F | 33 | 332605 | -3 | 0.000133 |
| 33 | 436130 | F | 33 | 460160 | -3 | 0.000133 |
| 30 | 12175 | P | 30 | 184239 | -2 | 0.000226 |
| 30 | 19265 | F | 30 | 73435 | -2 | 0.000226 |
| 30 | 36738 | P | 30 | 181921 | -2 | 0.000226 |
| 30 | 41542 | F | 30 | 445277 | -2 | 0.000226 |
| 30 | 54428 | P | 30 | 426995 | -2 | 0.000226 |
| 30 | 55176 | F | 30 | 64928 | -2 | 0.000226 |
| 30 | 62589 | P | 30 | 100133 | -2 | 0.000226 |
| 30 | 151444 | F | 30 | 298469 | -2 | 0.000226 |
| 30 | 158925 | F | 30 | 244673 | -2 | 0.000226 |
| 30 | 173626 | P | 30 | 396661 | -2 | 0.000226 |
| 30 | 206920 | F | 30 | 234607 | -2 | 0.000226 |
| 30 | 234607 | P | 30 | 377973 | -2 | 0.000226 |
| 30 | 244590 | F | 30 | 247146 | -2 | 0.000226 |
| 30 | 247361 | P | 30 | 294152 | -2 | 0.000226 |
| 30 | 332605 | F | 30 | 443571 | -2 | 0.000226 |
| 30 | 438619 | F | 30 | 468793 | -2 | 0.000226 |
| 30 | 443297 | F | 30 | 443329 | -2 | 0.000226 |
| 30 | 471930 | P | 30 | 483239 | -2 | 0.000226 |
| 32 | 19984 | P | 32 | 443261 | -3 | 0.000483 |
| 32 | 36725 | P | 32 | 181932 | -3 | 0.000483 |
| 32 | 55245 | F | 32 | 65011 | -3 | 0.000483 |
| 32 | 64926 | F | 32 | 216484 | -3 | 0.000483 |
| 32 | 64926 | P | 32 | 368407 | -3 | 0.000483 |
| 32 | 73872 | P | 32 | 443572 | -3 | 0.000483 |
| 32 | 74062 | P | 32 | 443387 | -3 | 0.000483 |
| 32 | 74071 | P | 32 | 443378 | -3 | 0.000483 |
| 32 | 74164 | P | 32 | 443258 | -3 | 0.000483 |
| 32 | 74587 | P | 32 | 234610 | -3 | 0.000483 |
| 32 | 74620 | P | 32 | 206890 | -3 | 0.000483 |
| 32 | 74620 | F | 32 | 378001 | -3 | 0.000483 |
| 32 | 81304 | F | 32 | 398822 | -3 | 0.000483 |
| 32 | 110684 | F | 32 | 110930 | -3 | 0.000483 |
| 32 | 113890 | P | 32 | 120144 | -3 | 0.000483 |
| 32 | 130341 | F | 32 | 243939 | -3 | 0.000483 |
| 32 | 232914 | F | 32 | 443264 | -3 | 0.000483 |
| 32 | 235038 | F | 32 | 443258 | -3 | 0.000483 |
| 32 | 235331 | F | 32 | 332605 | -3 | 0.000483 |
| 32 | 275793 | F | 32 | 398822 | -3 | 0.000483 |
| 32 | 360178 | P | 32 | 473334 | -3 | 0.000483 |
| 32 | 377524 | P | 32 | 383298 | -3 | 0.000483 |
| 32 | 393883 | F | 32 | 409311 | -3 | 0.000483 |
| 31 | 12173 | P | 31 | 105549 | -3 | 0.00175 |
| 31 | 12180 | P | 31 | 135425 | -3 | 0.00175 |
| 31 | 19488 | P | 31 | 233400 | -3 | 0.00175 |
| 31 | 21454 | P | 31 | 402360 | -3 | 0.00175 |
| 31 | 35080 | P | 31 | 296718 | -3 | 0.00175 |
| 31 | 52587 | F | 31 | 384466 | -3 | 0.00175 |
| 31 | 55177 | F | 31 | 216487 | -3 | 0.00175 |
| 31 | 55177 | P | 31 | 368405 | -3 | 0.00175 |
| 31 | 56408 | P | 31 | 290140 | -3 | 0.00175 |
| 31 | 73840 | P | 31 | 233220 | -3 | 0.00175 |
| 31 | 73865 | P | 31 | 235340 | -3 | 0.00175 |
| 31 | 74455 | P | 31 | 442973 | -3 | 0.00175 |
| 31 | 81262 | P | 31 | 297623 | -3 | 0.00175 |
| 31 | 104269 | P | 31 | 308631 | -3 | 0.00175 |
| 31 | 105546 | F | 31 | 484462 | -3 | 0.00175 |
| 31 | 112006 | F | 31 | 429510 | -3 | 0.00175 |
| 31 | 118109 | P | 31 | 180255 | -3 | 0.00175 |
| 31 | 131396 | F | 31 | 438401 | -3 | 0.00175 |
| 31 | 134346 | F | 31 | 182756 | -3 | 0.00175 |
| 31 | 150949 | C | 31 | 150950 | -3 | 0.00175 |
| 31 | 189596 | P | 31 | 189596 | -3 | 0.00175 |
| 31 | 202224 | F | 31 | 284508 | -3 | 0.00175 |
| 31 | 207648 | F | 31 | 208383 | -3 | 0.00175 |
| 31 | 207648 | P | 31 | 376509 | -3 | 0.00175 |
| 31 | 208383 | P | 31 | 377244 | -3 | 0.00175 |
| 31 | 224876 | P | 31 | 231414 | -3 | 0.00175 |
| 31 | 225266 | P | 31 | 334692 | -3 | 0.00175 |
| 31 | 232999 | F | 31 | 443381 | -3 | 0.00175 |
| 31 | 237190 | P | 31 | 264282 | -3 | 0.00175 |
| 31 | 263895 | P | 31 | 284507 | -3 | 0.00175 |
| 31 | 275131 | F | 31 | 447468 | -3 | 0.00175 |
| 31 | 366392 | F | 31 | 366410 | -3 | 0.00175 |
| 31 | 410568 | F | 31 | 466802 | -3 | 0.00175 |
| 31 | 417773 | F | 31 | 417807 | -3 | 0.00175 |
| 31 | 429967 | P | 31 | 478829 | -3 | 0.00175 |
| 30 | 1004 | F | 30 | 88830 | -3 | 0.00632 |
| 30 | 5343 | F | 30 | 274335 | -3 | 0.00632 |
| 30 | 12175 | P | 30 | 484464 | -3 | 0.00632 |
| 30 | 19670 | P | 30 | 332638 | -3 | 0.00632 |
| 30 | 28975 | F | 30 | 63789 | -3 | 0.00632 |
| 30 | 30792 | F | 30 | 438208 | -3 | 0.00632 |
| 30 | 54790 | F | 30 | 93610 | -3 | 0.00632 |
| 30 | 62983 | P | 30 | 460504 | -3 | 0.00632 |
| 30 | 73007 | P | 30 | 234034 | -3 | 0.00632 |
| 30 | 73899 | P | 30 | 233162 | -3 | 0.00632 |
| 30 | 74030 | P | 30 | 332450 | -3 | 0.00632 |
| 30 | 74551 | P | 30 | 206960 | -3 | 0.00632 |
| 30 | 74551 | F | 30 | 377933 | -3 | 0.00632 |
| 30 | 74626 | P | 30 | 442803 | -3 | 0.00632 |
| 30 | 80031 | P | 30 | 297690 | -3 | 0.00632 |
| 30 | 85357 | P | 30 | 181526 | -3 | 0.00632 |
| 30 | 99792 | F | 30 | 423985 | -3 | 0.00632 |
| 30 | 100618 | P | 30 | 466043 | -3 | 0.00632 |
| 30 | 105121 | F | 30 | 417425 | -3 | 0.00632 |
| 30 | 108397 | P | 30 | 336824 | -3 | 0.00632 |
| 30 | 110772 | F | 30 | 110810 | -3 | 0.00632 |
| 30 | 118954 | P | 30 | 179402 | -3 | 0.00632 |
| 30 | 120156 | F | 30 | 143355 | -3 | 0.00632 |
| 30 | 122291 | F | 30 | 291041 | -3 | 0.00632 |
| 30 | 134196 | F | 30 | 182607 | -3 | 0.00632 |
| 30 | 134337 | F | 30 | 182747 | -3 | 0.00632 |
| 30 | 135462 | F | 30 | 184270 | -3 | 0.00632 |
| 30 | 153505 | F | 30 | 427466 | -3 | 0.00632 |
| 30 | 161851 | F | 30 | 161890 | -3 | 0.00632 |
| 30 | 164698 | F | 30 | 181676 | -3 | 0.00632 |
| 30 | 179289 | P | 30 | 384341 | -3 | 0.00632 |
| 30 | 207054 | F | 30 | 442989 | -3 | 0.00632 |
| 30 | 207574 | F | 30 | 208310 | -3 | 0.00632 |
| 30 | 207574 | P | 30 | 376583 | -3 | 0.00632 |
| 30 | 208310 | P | 30 | 377319 | -3 | 0.00632 |
| 30 | 237269 | P | 30 | 264216 | -3 | 0.00632 |
| 30 | 248804 | F | 30 | 318165 | -3 | 0.00632 |
| 30 | 249463 | P | 30 | 340505 | -3 | 0.00632 |
| 30 | 257588 | F | 30 | 297980 | -3 | 0.00632 |
| 30 | 264173 | P | 30 | 408195 | -3 | 0.00632 |
| 30 | 264173 | F | 30 | 429450 | -3 | 0.00632 |
| 30 | 264585 | F | 30 | 264633 | -3 | 0.00632 |
| 30 | 287412 | P | 30 | 425556 | -3 | 0.00632 |
| 30 | 306690 | F | 30 | 306731 | -3 | 0.00632 |
| 30 | 315634 | P | 30 | 466043 | -3 | 0.00632 |
| 30 | 348003 | P | 30 | 438780 | -3 | 0.00632 |
| 30 | 376584 | F | 30 | 377320 | -3 | 0.00632 |
| 30 | 377839 | P | 30 | 442989 | -3 | 0.00632 |
| 30 | 383529 | F | 30 | 443580 | -3 | 0.00632 |
| 30 | 390255 | P | 30 | 390570 | -3 | 0.00632 |
| 30 | 410577 | F | 30 | 466811 | -3 | 0.00632 |
| 30 | 417425 | P | 30 | 433548 | -3 | 0.00632 |
| 30 | 435627 | F | 30 | 459641 | -3 | 0.00632 |
| 30 | 437663 | F | 30 | 461296 | -3 | 0.00632 |
| 30 | 438605 | F | 30 | 442598 | -3 | 0.00632 |

Supplementary Table 6 The homologous DNA fragment in the *Ficus hirta* mitochondrial genome.

| Number | Mitochondrial genome | % Identity | Alignment Length | Mismatches | Gap Openings | Alignment start (chloroplast genome) | Alignment end (chloroplast genome) | Alignment start (mitochondrial genome) | Alignment end (mitochondrial genome) | E-value | Bit Score | MTPT annotation |
| --- | --- | --- | --- | --- | --- | --- | --- | --- | --- | --- | --- | --- |
| MTPT1 | mtDNA | 100 | 29 | 0 | 0 | 11314 | 11342 | 160167 | 160139 | 2.71E-06 | 54.7 | Partial (*atp*A) |
| MTPT2 | mtDNA | 100 | 33 | 0 | 0 | 42670 | 42702 | 343303 | 343271 | 1.62E-08 | 62.1 | Partial (*psa*A) |
| MTPT3 | mtDNA | 99.576 | 236 | 1 | 0 | 97299 | 97534 | 340839 | 341074 | 1.07E-119 | 431 | Partial (*ycf*2) |
|  | mtDNA | 99.576 | 236 | 1 | 0 | 151223 | 151458 | 341074 | 340839 | 1.07E-119 | 431 | Partial (*ycf*2) |
| MTPT4 | mtDNA | 98.78 | 82 | 0 | 1 | 32341 | 32422 | 463430 | 463350 | 1.57E-33 | 145 | Complete (*trn*D-GUC) |
| MTPT5 | mtDNA | 98.765 | 81 | 1 | 0 | 107062 | 107142 | 426463 | 426383 | 1.57E-33 | 145 | Partial (*trn*I-GAU) |
|  | mtDNA | 98.765 | 81 | 1 | 0 | 141615 | 141695 | 426383 | 426463 | 1.57E-33 | 145 | Partial (*trn*I-GAU) |
| MTPT6 | mtDNA | 98.413 | 63 | 1 | 0 | 107589 | 107651 | 77201 | 77139 | 1.59E-23 | 111 | Partial (*trn*A-UGC) |
|  | mtDNA | 98.413 | 63 | 1 | 0 | 141106 | 141168 | 77139 | 77201 | 1.59E-23 | 111 | Partial (*trn*A-UGC) |
| MTPT7 | mtDNA | 97.709 | 1266 | 27 | 2 | 58751 | 60014 | 223875 | 225140 | 0 | 2176 | Partial (*rbc*L) |
| MTPT8 | mtDNA | 96.229 | 663 | 6 | 2 | 42791 | 43453 | 343293 | 343936 | 0 | 1068 | Partial (*psa*A) |
| MTPT9 | mtDNA | 95.098 | 612 | 17 | 8 | 11448 | 12049 | 165387 | 164779 | 0 | 952 | Partial (*atp*A) |
| MTPT10 | mtDNA | 94.937 | 79 | 4 | 0 | 55516 | 55594 | 166333 | 166255 | 2.04E-27 | 124 | Complete (*trn*M-CAU) |
| MTPT11 | mtDNA | 91.892 | 148 | 11 | 1 | 36970 | 37116 | 160889 | 161036 | 7.08E-52 | 206 | Partial (*psb*C) |
| MTPT12 | mtDNA | 91.497 | 541 | 19 | 4 | 823 | 1363 | 379292 | 378779 | 0 | 719 | Partial (*psb*A) |
| MTPT13 | mtDNA | 91.231 | 536 | 18 | 5 | 825 | 1360 | 205636 | 206142 | 0 | 702 | Partial (*psb*A) |
| MTPT14 | mtDNA | 88.145 | 523 | 53 | 3 | 130025 | 130547 | 107657 | 107144 | 9.93E-175 | 614 | Partial (*ycf*1) |
| MTPT15 | mtDNA | 81.609 | 87 | 4 | 6 | 90558 | 90632 | 480846 | 480760 | 1.62E-08 | 62.1 | Complete (*trn*I-CAU) |
|  | mtDNA | 81.609 | 87 | 4 | 6 | 158125 | 158199 | 480760 | 480846 | 1.62E-08 | 62.1 | Complete (*trn*I-CAU) |

Supplementary Table 7 RNA editing

| gene | Base position | Amino position | Codon change | Amino change | Left motifs | Edited site | Right motifs | Probability |
| --- | --- | --- | --- | --- | --- | --- | --- | --- |
| atp4 | 59 | 20 | UCU->UUU | Ser->Phe | GCTATTTGCTGCTATTCTAT | C | TATTTGTGCATTAAGTTCGA | 0.995 |
| atp4 | 89 | 30 | UCA->UUA | Ser->Leu | ATTAAGTTCGAAGAAGATCT | C | AATCTATAATGAAGAAATGA | 1 |
| atp4 | 250 | 84 | CUG->UUG | Leu->Leu | ATCCTAACGAAGTAGTTCTT | C | TGGAATCCAATGAACAACAA | 0.956 |
| atp4 | 395 | 132 | UCA->UUA | Ser->Leu | CCGAAACCTAAATGTTAAGT | C | AGCAACACTTCCAAATGCCA | 0.999 |
| atp4 | 407 | 136 | CCA->CUA | Pro->Leu | TGTTAAGTCAGCAACACTTC | C | AAATGCCACTTCTTCCCGTC | 0.993 |
| atp4 | 416 | 139 | ACU->AUU | Thr->Ile | AGCAACACTTCCAAATGCCA | C | TTCTTCCCGTCGCATCCGTC | 0.987 |
| atp9 | 20 | 7 | UCA->UUA | Ser->Leu | AATGTTAGAAGGTGCAAAAT | C | AATGGGTGCCGGAGCTGCTA | 1 |
| atp9 | 50 | 17 | UCA->UUA | Ser->Leu | CGGAGCTGCTACAATTGCTT | C | AGCGGGAGCTGCTGTCGGTA | 1 |
| atp9 | 134 | 45 | UCA->UUA | Ser->Leu | TCCATCATTGGCTAAACAAT | C | ATTTGGTTATGCCATTTTGG | 1 |
| atp9 | 182 | 61 | UCG->UUG | Ser->Leu | TCTAACCGAAGCTATTGCAT | C | GTTTGCCCCAATGATGGCCT | 1 |
| atp9 | 191 | 64 | CCA->CUA | Pro->Leu | AGCTATTGCATCGTTTGCCC | C | AATGATGGCCTTTCTGATCT | 1 |
| atp9 | 205 | 69 | CUG->UUG | Leu->Leu | TTGCCCCAATGATGGCCTTT | C | TGATCTCATCCGTATTCCAA | 0.902 |
| atp9 | 212 | 71 | UCA->UUA | Ser->Leu | AATGATGGCCTTTCTGATCT | C | ATCCGTATTCCAATCGAAGA | 0.994 |
| atp9 | 215 | 72 | UCC->UUC | Ser->Phe | GATGGCCTTTCTGATCTCAT | C | CGTATTCCAATCGAAGAAAG | 0.99 |
| atp9 | 223 | 75 | CGA->UGA | Arg->End | TTCTGATCTCATCCGTATTC | C | AATCGAAGAAAGAAGGTTTC | 0.9 |
| ccmB | 28 | 10 | CAU->UAU | His->Tyr | GACTCTTTCTTGAACTATAT | C | ATAAACAGATCTTCCCCTCC | 0.988 |
| ccmB | 43 | 15 | CCC->UCC | Pro->Ser | TATATCATAAACAGATCTTC | C | CCTCCACACCAATCACGAGT | 0.995 |
| ccmB | 71 | 24 | CCA->CUA | Pro->Leu | ACCAATCACGAGTTTTTCTC | C | ATTCCTCTCGTATATCGTCG | 0.992 |
| ccmB | 80 | 27 | UCG->UUG | Ser->Leu | GAGTTTTTCTCCATTCCTCT | C | GTATATCGTCGTAACGCCCT | 0.969 |
| ccmB | 87 | 29 | AUC->AUU | Ile->Ile | TCTCCATTCCTCTCGTATAT | C | GTCGTAACGCCCTTAATGCT | 0.963 |
| ccmB | 128 | 43 | UCA->UUA | Ser->Leu | AGGTTTTGAAAAAGACTTTT | C | ATGTCATTCCCATTTAGGTC | 0.998 |
| ccmB | 137 | 46 | UCC->UUC | Ser->Phe | AAAAGACTTTTCATGTCATT | C | CCATTTAGGTCCGATTCGGA | 0.988 |
| ccmB | 148 | 50 | CCG->UUG | Pro->Leu | CATGTCATTCCCATTTAGGT | C | CGATTCGGATCCCTCCGTTG | 0.989 |
| ccmB | 149 | 50 | CCG->UUG | Pro->Leu | ATGTCATTCCCATTTAGGTC | C | GATTCGGATCCCTCCGTTGT | 0.996 |
| ccmB | 154 | 52 | CGG->UGG | Arg->Trp | ATTCCCATTTAGGTCCGATT | C | GGATCCCTCCGTTGTTTCCT | 0.978 |
| ccmB | 160 | 54 | CCU->UCU | Pro->Ser | ATTTAGGTCCGATTCGGATC | C | CTCCGTTGTTTCCTTTTCCT | 0.992 |
| ccmB | 164 | 55 | CCG->CUG | Pro->Leu | AGGTCCGATTCGGATCCCTC | C | GTTGTTTCCTTTTCCTCCTG | 0.991 |
| ccmB | 172 | 58 | CCU->UCU | Pro->Ser | TTCGGATCCCTCCGTTGTTT | C | CTTTTCCTCCTGCACCTTTT | 0.961 |
| ccmB | 181 | 61 | CCU->UCU | Pro->Ser | CTCCGTTGTTTCCTTTTCCT | C | CTGCACCTTTTCCTCGAAAT | 0.987 |
| ccmB | 193 | 65 | CCU->UUU | Pro->Phe | CTTTTCCTCCTGCACCTTTT | C | CTCGAAATGAGAAAGAAGAT | 0.944 |
| ccmB | 194 | 65 | CCU->UUU | Pro->Phe | TTTTCCTCCTGCACCTTTTC | C | TCGAAATGAGAAAGAAGATG | 0.994 |
| ccmB | 304 | 102 | CGU->UGU | Arg->Cys | ACCGGGTTATTAAAATAAGT | C | GTGTTTTCTGTGGTTTTCCC | 0.954 |
| ccmB | 367 | 123 | CGG->UGG | Arg->Trp | TCGGTCGATCCGGAATGGAT | C | GGTTAAACATTCTATTAGGG | 0.982 |
| ccmB | 379 | 127 | CUA->UUA | Leu->Leu | GAATGGATCGGTTAAACATT | C | TATTAGGGAGCCTGGTCTTG | 0.932 |
| ccmB | 424 | 142 | CGU->UGU | Arg->Cys | TTCTGTGTGGTATTCATTCT | C | GTTCGGCTCTTGGAATCACA | 0.994 |
| ccmB | 428 | 143 | UCG->UUG | Ser->Leu | GTGTGGTATTCATTCTCGTT | C | GGCTCTTGGAATCACATCCA | 0.996 |
| ccmB | 475 | 159 | CCA->UUA | Pro->Leu | GTTGTAACAGCTCGCAAAAT | C | CAACCACTTTACCTACTTCA | 0.92 |
| ccmB | 476 | 159 | CCA->UUA | Pro->Leu | TTGTAACAGCTCGCAAAATC | C | AACCACTTTACCTACTTCAT | 0.945 |
| ccmB | 494 | 165 | UCA->UUA | Ser->Leu | TCCAACCACTTTACCTACTT | C | ATTGCCCCCAACCCTTTCTT | 0.983 |
| ccmB | 502 | 168 | CCA->UUA | Pro->Leu | CTTTACCTACTTCATTGCCC | C | CAACCCTTTCTTGTACCTCT | 0.949 |
| ccmB | 503 | 168 | CCA->UUA | Pro->Leu | TTTACCTACTTCATTGCCCC | C | AACCCTTTCTTGTACCTCTA | 0.993 |
| ccmB | 512 | 171 | UCU->UUU | Ser->Phe | TTCATTGCCCCCAACCCTTT | C | TTGTACCTCTATTGAAACAG | 0.976 |
| ccmB | 548 | 183 | CCU->CUU | Pro->Leu | AACAGAATGGTTTCATGTTC | C | TTCATCGATTGGTTATTCCT | 1 |
| ccmB | 551 | 184 | UCA->UUA | Ser->Leu | AGAATGGTTTCATGTTCCTT | C | ATCGATTGGTTATTCCTCTC | 0.996 |
| ccmB | 554 | 185 | UCG->UUG | Ser->Leu | ATGGTTTCATGTTCCTTCAT | C | GATTGGTTATTCCTCTCCGT | 0.991 |
| ccmB | 566 | 189 | UCC->UUC | Ser->Phe | TCCTTCATCGATTGGTTATT | C | CTCTCCGTTCGTATCTCTTT | 0.991 |
| ccmB | 569 | 190 | UCU->UUU | Ser->Phe | TTCATCGATTGGTTATTCCT | C | TCCGTTCGTATCTCTTTTTC | 0.979 |
| ccmB | 572 | 191 | CCG->CUG | Pro->Leu | ATCGATTGGTTATTCCTCTC | C | GTTCGTATCTCTTTTTCCAA | 0.999 |
| ccmB | 596 | 199 | UCG->UUG | Ser->Leu | CGTATCTCTTTTTCCAATTT | C | GGTCTCGATTAGTTTACAAG | 0.999 |
| ccmC | 5 | 2 | UCC->UUC | Ser->Phe | AGGACAAAATTCTCACATGT | C | CGTTTCGTTATTACAACCTT | 0.988 |
| ccmC | 76 | 26 | CGG->UGG | Arg->Trp | CGCAAATTCTCATTGGATCT | C | GGTTGTTCTTAACAGCGATG | 1 |
| ccmC | 103 | 35 | CAU->UAU | His->Tyr | TCTTAACAGCGATGGCTATT | C | ATTTAAGTCTTCGGGTAGCA | 0.999 |
| ccmC | 115 | 39 | CGG->UGG | Arg->Trp | TGGCTATTCATTTAAGTCTT | C | GGGTAGCACCACTAGATCTT | 0.991 |
| ccmC | 133 | 45 | CUU->UUU | Leu->Phe | TTCGGGTAGCACCACTAGAT | C | TTCAACAAGGTGGAAATTCT | 0.987 |
| ccmC | 161 | 54 | CCG->CUG | Pro->Leu | AGGTGGAAATTCTCGTATTC | C | GTATGTACATGTTCCTGCGG | 1 |
| ccmC | 179 | 60 | GCG->GUG | Ala->Val | TCCGTATGTACATGTTCCTG | C | GGCTCGGATGAGTATTCTTG | 0.999 |
| ccmC | 184 | 62 | CGG->UGG | Arg->Trp | ATGTACATGTTCCTGCGGCT | C | GGATGAGTATTCTTGTTTAT | 0.994 |
| ccmC | 281 | 94 | ACA->AUA | Thr->Ile | TCGCTCTTCCGGAACCGGTA | C | AGAAATGGGTGCTTTTTTTA | 0.986 |
| ccmC | 331 | 111 | CGG->UGG | Arg->Trp | CCTTAGTTACTGGGGGGTTT | C | GGGGAAGACCTATGTGGGGC | 0.999 |
| ccmC | 395 | 132 | UCG->UUG | Ser->Leu | TTTAACCTCTGTATTCATCT | C | GTTCCTTATTTACCTGGGTG | 0.996 |
| ccmC | 399 | 133 | UUC->UUU | Phe->Phe | ACCTCTGTATTCATCTCGTT | C | CTTATTTACCTGGGTGCACT | 0.994 |
| ccmC | 400 | 134 | CUU->UUU | Leu->Phe | CCTCTGTATTCATCTCGTTC | C | TTATTTACCTGGGTGCACTG | 0.999 |
| ccmC | 421 | 141 | CGU->UGU | Arg->Cys | TTATTTACCTGGGTGCACTG | C | GTTTTCAAAAGCTTCCTGTC | 0.999 |
| ccmC | 436 | 146 | CCU->UCU | Pro->Ser | CACTGCGTTTTCAAAAGCTT | C | CTGTCGAACCGGCTTCTATT | 0.999 |
| ccmC | 446 | 149 | CCG->CUG | Pro->Leu | TCAAAAGCTTCCTGTCGAAC | C | GGCTTCTATTTCAATCCGTG | 0.996 |
| ccmC | 458 | 153 | UCA->UUA | Ser->Leu | TGTCGAACCGGCTTCTATTT | C | AATCCGTGCTGGACCGATCG | 0.979 |
| ccmC | 463 | 155 | CGU->UGU | Arg->Cys | AACCGGCTTCTATTTCAATC | C | GTGCTGGACCGATCGATATA | 0.999 |
| ccmC | 467 | 156 | GCU->GUU | Ala->Val | GGCTTCTATTTCAATCCGTG | C | TGGACCGATCGATATACCAA | 0.997 |
| ccmC | 473 | 158 | CCG->CUG | Pro->Leu | TATTTCAATCCGTGCTGGAC | C | GATCGATATACCAATAATTA | 0.991 |
| ccmC | 497 | 166 | UCU->UUU | Ser->Phe | CGATATACCAATAATTAAGT | C | TTCAGTCAACTGGTGGAATA | 0.999 |
| ccmC | 521 | 174 | UCG->UUG | Ser->Leu | AGTCAACTGGTGGAATACAT | C | GCATCAACCCGGGAGCATTA | 0.996 |
| ccmC | 548 | 183 | UCU->UUU | Ser->Phe | ACCCGGGAGCATTAGCCGAT | C | TGGTACATCAATACATGTTC | 0.985 |
| ccmC | 568 | 190 | CCU->UCU | Pro->Ser | CTGGTACATCAATACATGTT | C | CTATGCCCATTCCAATCTTG | 0.999 |
| ccmC | 575 | 192 | CCC->CUC | Pro->Leu | ATCAATACATGTTCCTATGC | C | CATTCCAATCTTGTCTAACT | 0.998 |
| ccmC | 605 | 202 | UCC->UUC | Ser->Phe | CTTGTCTAACTTTGCTAACT | C | CCCCTTCTCAACCCGTATCT | 1 |
| ccmC | 608 | 203 | CCC->CUC | Pro->Leu | GTCTAACTTTGCTAACTCCC | C | CTTCTCAACCCGTATCTTCT | 0.998 |
| ccmC | 614 | 205 | UCA->UUA | Ser->Leu | CTTTGCTAACTCCCCCTTCT | C | AACCCGTATCTTCTTTGTTC | 0.99 |
| ccmC | 650 | 217 | CCU->CUU | Pro->Leu | TGTTCTGGAAACACGTCTTC | C | TATTCCATCTTTTCCCGAAT | 0.996 |
| ccmC | 656 | 219 | CCA->CUA | Pro->Leu | GGAAACACGTCTTCCTATTC | C | ATCTTTTCCCGAATTTCCTT | 0.992 |
| ccmC | 665 | 222 | CCC->CUC | Pro->Leu | TCTTCCTATTCCATCTTTTC | C | CGAATTTCCTTTAACGGAAG | 0.999 |
| ccmFC | 38 | 13 | UCC->UUC | Ser->Phe | CTCTTTCTTTTTCATTACTT | C | CATGGTCGTGCCTTGTGGCA | 0.995 |
| ccmFC | 50 | 17 | CCU->CUU | Pro->Leu | CATTACTTCCATGGTCGTGC | C | TTGTGGCACGGCAGCACCCG | 1 |
| ccmFC | 103 | 35 | CCC->UCC | Pro->Ser | GGTTCGTCAGTAGAGATGTT | C | CCACAGGTGCCCCTTCTTCC | 0.999 |
| ccmFC | 119 | 40 | UCU->UUU | Ser->Phe | TGTTCCCACAGGTGCCCCTT | C | TTCCAATGGTACTATAATTC | 0.987 |
| ccmFC | 122 | 41 | UCC->UUC | Ser->Phe | TCCCACAGGTGCCCCTTCTT | C | CAATGGTACTATAATTCCTA | 1 |
| ccmFC | 146 | 49 | CCU->CUU | Pro->Leu | TGGTACTATAATTCCTATTC | C | TATCCCTGCATTCCCTCTTT | 0.996 |
| ccmFC | 151 | 51 | CCU->UCU | Pro->Ser | CTATAATTCCTATTCCTATC | C | CTGCATTCCCTCTTTTTGTC | 0.962 |
| ccmFC | 304 | 102 | CGU->UGU | Arg->Cys | AAACTAGAAACGCTTTATTT | C | GTTTCGTTCTCGTTCTTCAT | 0.978 |
| ccmFC | 385 | 129 | CGU->UGU | Arg->Cys | AATCTTTCTGCGGTGTGCTC | C | GTTTACTATTCTTTCGTACT | 0.999 |
| ccmFC | 400 | 134 | CGU->UGU | Arg->Cys | TGCTCCGTTTACTATTCTTT | C | GTACTCTCTTCTCTTTAGCA | 0.998 |
| ccmFC | 872 | 291 | UCU->UUU | Ser->Phe | TCATTTACATGGACCCACTT | C | TCATTCCATTTGTGGGAATT | 0.997 |
| ccmFC | 1154 | 385 | UCG->UUG | Ser->Leu | ATTTACGGATCTATATGCTT | C | GATTGGAACTGGAAGTTCCA | 0.999 |
| ccmFC | 1228 | 410 | CGG->UGG | Arg->Trp | TGCCTTTTCTTTTTTTTATT | C | GGATAGGATTTATGTTGGCT | 0.967 |
| ccmFC | 1253 | 418 | UCG->UUG | Ser->Leu | AGGATTTATGTTGGCTTCGT | C | GGGAGGCTCGCGTAGTTTGT | 0.995 |
| ccmFC | 1262 | 421 | UCG->UUG | Ser->Leu | GTTGGCTTCGTCGGGAGGCT | C | GCGTAGTTTGTTACGTCAGC | 0.983 |
| ccmFC | 1309 | 437 | CGA->UGA | Arg->End | AGGATAAGTTGCGTTGGAAT | C | GAGAAAGTTCCGTGGAGTTC | 0.998 |
| ccmFN | 38 | 13 | CCG->CUG | Pro->Leu | GTTTCATTATTCGTTATTTC | C | GGGTCTTTTCGTTGCATTCA | 0.99 |
| ccmFN | 98 | 33 | CCU->CUU | Pro->Leu | ACCTGCGTTTGGTGCAGCAC | C | TGCATTTTGGTGCATTCTTC | 1 |
| ccmFN | 137 | 46 | UCG->UUG | Ser->Leu | TCTTTCTTTCCTTGGTCTTT | C | GTTCCGTCATATTCCTAATA | 0.981 |
| ccmFN | 142 | 48 | CGU->UGU | Arg->Cys | CTTTCCTTGGTCTTTCGTTC | C | GTCATATTCCTAATAACTTA | 0.987 |
| ccmFN | 151 | 51 | CCU->UCU | Pro->Ser | GTCTTTCGTTCCGTCATATT | C | CTAATAACTTATCCAATTAC | 0.99 |
| ccmFN | 248 | 83 | UCA->UUA | Ser->Leu | TCATGAGGGTAGTATTTTAT | C | ATGGTGTCGGATCCCAAGTT | 0.999 |
| ccmFN | 256 | 86 | CGG->UGG | Arg->Trp | GTAGTATTTTATCATGGTGT | C | GGATCCCAAGTTTTTATGGA | 0.999 |
| ccmFN | 263 | 88 | CCA->CUA | Pro->Leu | TTTATCATGGTGTCGGATCC | C | AAGTTTTTATGGATTCCTTC | 1 |
| ccmFN | 283 | 95 | CUU->UUU | Leu->Phe | CAAGTTTTTATGGATTCCTT | C | TTTGTTACCGGGGTCGACCC | 0.995 |
| ccmFN | 365 | 122 | UCG->UUG | Ser->Leu | TTTTTTTTATTCCTTTGTCT | C | GAACTTCGTGAAGAACTCCA | 0.928 |
| ccmFN | 372 | 124 | UUC->UUU | Phe->Phe | TATTCCTTTGTCTCGAACTT | C | GTGAAGAACTCCATTCTATC | 0.989 |
| ccmFN | 707 | 236 | CCU->CUU | Pro->Leu | CATTGCTTTGTTTTTTTCTC | C | TTTCCTATCAGCGAGTTCCG | 1 |
| ccmFN | 716 | 239 | UCA->UUA | Ser->Leu | GTTTTTTTCTCCTTTCCTAT | C | AGCGAGTTCCGATCCTTTTG | 0.994 |
| ccmFN | 754 | 252 | CGU->UGU | Arg->Cys | TTGTTCGAAATTTCTTCGTT | C | GTACCGAACCGCTTGCAGAA | 0.998 |
| ccmFN | 776 | 259 | UCA->UUA | Ser->Leu | TACCGAACCGCTTGCAGAAT | C | AAATCCTGTTCCACAAGATC | 0.981 |
| ccmFN | 788 | 263 | CCA->CUA | Pro->Leu | TGCAGAATCAAATCCTGTTC | C | ACAAGATCCTATATCAGCTA | 0.999 |
| ccmFN | 803 | 268 | UCA->UUA | Ser->Leu | TGTTCCACAAGATCCTATAT | C | AGCTATACATCCTCCTTGCA | 0.991 |
| ccmFN | 952 | 318 | CGC->UGC | Arg->Cys | AAAAGAATGGAACGCTGCTT | C | GCTCTGCTGGATGCGTTGGA | 0.988 |
| ccmFN | 1270 | 424 | CGG->UGG | Arg->Trp | TAATTTGGATCTTGACATGT | C | GGTGGTTTTTAACCGTAGGC | 0.996 |
| ccmFN | 1298 | 433 | CCA->CUA | Pro->Leu | TTTAACCGTAGGCATCTTGC | C | AGGAAGTTGGTGGGCTCATC | 1 |
| ccmFN | 1315 | 439 | CAU->UAU | His->Tyr | TGCCAGGAAGTTGGTGGGCT | C | ATCATGAATTAGGTCGGGGT | 1 |
| ccmFN | 1330 | 444 | CGG->UGG | Arg->Trp | GGGCTCATCATGAATTAGGT | C | GGGGTGGCTGGTGGTTTCGG | 0.999 |
| ccmFN | 1348 | 450 | CGG->UGG | Arg->Trp | GTCGGGGTGGCTGGTGGTTT | C | GGGATCCCGTAGAAAATGCT | 0.999 |
| ccmFN | 1381 | 461 | CGG->UGG | Arg->Trp | AAAATGCTTCTTTTATGCCT | C | GGGTATTAGCCACAGCTCGT | 1 |
| ccmFN | 1399 | 467 | CGU->UGU | Arg->Cys | CTCGGGTATTAGCCACAGCT | C | GTATTCATTCAGTAATTCTA | 0.996 |
| ccmFN | 1442 | 481 | UCC->UUC | Ser->Phe | CCTTCGTCATTCTTGTACCT | C | CCTTCTGAATATTGTGACTC | 0.996 |
| ccmFN | 1462 | 488 | CUU->UUU | Leu->Phe | CCCTTCTGAATATTGTGACT | C | TTCCCTGCTGTGTCTCAGGA | 0.988 |
| ccmFN | 1478 | 493 | UCA->UUA | Ser->Leu | GACTCTTCCCTGCTGTGTCT | C | AGGAACCTTTTCAATACGGT | 1 |
| ccmFN | 1513 | 505 | CCC->UCC | Pro->Ser | TACGGTCCGGATTGCTAGCT | C | CCGTTCATAGTTTTGCTACA | 0.999 |
| cob | 47 | 16 | UCA->UUA | Ser->Leu | TATTCTTAAAGAACCTATAT | C | ATCCACACTTAATCAGCATT | 0.906 |
| cob | 178 | 60 | CAC->UAC | His->Tyr | GCGTTTTTTTAGCTATGCAT | C | ACACACCTCATGTGGATCTA | 0.999 |
| cob | 286 | 96 | CUC->UUC | Leu->Phe | CTAATGGGGCAAGTATGTTT | C | TCATTGTGGTTCACCTACAT | 1 |
| cob | 298 | 100 | CAC->UAC | His->Tyr | GTATGTTTCTCATTGTGGTT | C | ACCTACATATTTTTCGTGGT | 0.997 |
| cob | 325 | 109 | CAU->UAU | His->Tyr | ATATTTTTCGTGGTCTATAT | C | ATGCGAGTTATAGCAGTCCT | 1 |
| cob | 407 | 136 | ACA->AUA | Thr->Ile | AATGATTGTGACAGCTTTTA | C | AGGATACGTACTACCTTGGG | 0.999 |
| cob | 737 | 246 | UCU->UUU | Ser->Phe | GGTAGCTTTTGCTATCTTTT | C | TTCCATTTGGATTTTTTATG | 0.995 |
| cob | 853 | 285 | CAU->UAU | His->Tyr | AATGGTATTTCCTACCGATC | C | ATGCCATTCTTCGTAGTATA | 0.999 |
| cob | 908 | 303 | CCA->CUA | Pro->Leu | AGGTGTAGCCGCAATAGCAC | C | AGTTTTTATATGTCTGTTGG | 0.998 |
| cob | 982 | 328 | CAC->UAC | His->Tyr | GTTCAAGTTTTCGCCCTATT | C | ACCAAGGAATATTTTGGTTG | 0.987 |
| cob | 1084 | 362 | CCU->UCU | Pro->Ser | TTACTATAGGACAAATTTCT | C | CTTTTCTTTTCTTCTTGTTC | 0.999 |
| cob | 1124 | 375 | CCG->CUG | Pro->Leu | CTTTGCCATAACGCCCATTC | C | GGGACAAGTTGGAAGAGGAA | 0.996 |
| cox2 | 721 | 241 | CCU->UCU | Pro->Ser | CTATCGTCGTAGAAGCTGTT | C | CTAGGAAAGATTATGGTTCT | 0.999 |
| cox2 | 742 | 248 | CGG->UGG | Arg->Trp | CTAGGAAAGATTATGGTTCT | C | GGGTATCCAATCAATTAATC | 0.999 |
| matR | 193 | 65 | CCA->UCA | Pro->Ser | TCGACCGACATCGACTCATC | C | CAATCTTTAAGGAAGAGATC | 0.994 |
| matR | 326 | 109 | CCA->CUA | Pro->Leu | TGTACTACTATCGGCCCTAC | C | AGGCAACATCTACCTACACA | 0.995 |
| matR | 413 | 138 | UCG->UUG | Ser->Leu | GATTGTTCAGAGAATCAGAT | C | GGTTCTATTAAGGACAGGTC | 0.973 |
| matR | 923 | 308 | UCA->UUA | Ser->Leu | CGCGCGATATGCCGACGACT | C | ACTACTGGGAATCGTGGGTG | 1 |
| matR | 1064 | 355 | CCC->CUC | Pro->Leu | ACGGAGTACGGTAGAATTCC | C | CGGTACGGTCATTCGGGAAG | 0.999 |
| matR | 1679 | 560 | UCC->UUC | Ser->Phe | CGGAGACATCGTAAATTGGT | C | CGCGGGCATCGCGATAAGTC | 0.996 |
| matR | 1700 | 567 | CCU->CUU | Pro->Leu | CGCGGGCATCGCGATAAGTC | C | TCTGTCCTACTACAGGTGCC | 0.999 |
| matR | 1720 | 574 | CGC->UGC | Arg->Cys | CTCTGTCCTACTACAGGTGC | C | GCGACAACCTTTACCAAGTC | 1 |
| matR | 1734 | 578 | UAC->UAU | Tyr->Tyr | AGGTGCCGCGACAACCTTTA | C | CAAGTCCGAACGATTGTCGA | 0.996 |
| matR | 1756 | 586 | CAC->UAC | His->Tyr | AAGTCCGAACGATTGTCGAC | C | ACCAGATCCGCTGGTCTGCA | 0.995 |
| matR | 1826 | 609 | CCA->CUA | Pro->Leu | CTCGGCGCGGAATATAATCC | C | AAAGTACTCCAAAGACTCAA | 0.998 |
| matR | 1844 | 615 | UCA->UUA | Ser->Leu | CCCAAAGTACTCCAAAGACT | C | AAATATAGTAAATCAAGAAG | 0.999 |
| mttB | 16 | 6 | CAU->UAU | His->Tyr | CATATATATCCTATGAATTT | C | ATTTCGCACCGGAAACTATT | 0.985 |
| mttB | 25 | 9 | CCG->UUG | Pro->Leu | CCTATGAATTTCATTTCGCA | C | CGGAAACTATTCTAGGAGAA | 0.987 |
| mttB | 26 | 9 | CCG->UUG | Pro->Leu | CTATGAATTTCATTTCGCAC | C | GGAAACTATTCTAGGAGAAG | 0.985 |
| mttB | 59 | 20 | UCC->UUC | Ser->Phe | AGGAGAAGTTCGAATCCGTT | C | CGTTCGGATATTGATCGGTC | 0.946 |
| mttB | 64 | 22 | CGG->UGG | Arg->Trp | AAGTTCGAATCCGTTCCGTT | C | GGATATTGATCGGTCTTGGT | 0.987 |
| mttB | 100 | 34 | CGU->UGU | Arg->Cys | TTGGTTTGACATGGTTTACG | C | GTTACTGGTTCCCGGAAGAG | 0.999 |
| mttB | 112 | 38 | CCG->UCG | Pro->Ser | GGTTTACGCGTTACTGGTTC | C | CGGAAGAGTTAATATCTCCA | 0.998 |
| mttB | 128 | 43 | UCU->UUU | Ser->Phe | GTTCCCGGAAGAGTTAATAT | C | TCCATTAGCTAAACCCTTTC | 0.996 |
| mttB | 131 | 44 | CCA->CUA | Pro->Leu | CCCGGAAGAGTTAATATCTC | C | ATTAGCTAAACCCTTTCTTA | 0.985 |
| mttB | 178 | 60 | CGU->UGU | Arg->Cys | CTTTGGACTCGTATTTTGTT | C | GTACACAATCAACGGAGGCC | 0.998 |
| mttB | 188 | 63 | UCA->UUA | Ser->Leu | GTATTTTGTTCGTACACAAT | C | AACGGAGGCCTCCCCGACAT | 1 |
| mttB | 200 | 67 | UCC->UUC | Ser->Phe | TACACAATCAACGGAGGCCT | C | CCCGACATATGTTGCAACGT | 1 |
| mttB | 202 | 68 | CCG->UCG | Pro->Ser | CACAATCAACGGAGGCCTCC | C | CGACATATGTTGCAACGTCT | 0.999 |
| mttB | 252 | 84 | CCC->CCU | Pro->Pro | TGCTCTTATTTCGTCTTTCC | C | TTAATAAGTCATCAAATTTG | 0.926 |
| mttB | 262 | 88 | CAU->UAU | His->Tyr | TCGTCTTTCCCTTAATAAGT | C | ATCAAATTTGGTGCTTTTTG | 0.991 |
| mttB | 328 | 110 | CUC->UUC | Leu->Phe | GGACGAAATACAATCGATTC | C | TCTATTTAAGTGGTTCTCGC | 0.999 |
| mttB | 344 | 115 | UCU->UUU | Ser->Phe | ATTCCTCTATTTAAGTGGTT | C | TCGCTTCTCCTTGTTCCTGT | 0.967 |
| mttB | 346 | 116 | CGC->UGC | Arg->Cys | TCCTCTATTTAAGTGGTTCT | C | GCTTCTCCTTGTTCCTGTTC | 0.999 |
| mttB | 373 | 125 | CCU->UUU | Pro->Phe | CCTTGTTCCTGTTCCTAACT | C | CTCCCCGGGTAGTTCCCAAT | 0.948 |
| mttB | 374 | 125 | CCU->UUU | Pro->Phe | CTTGTTCCTGTTCCTAACTC | C | TCCCCGGGTAGTTCCCAATG | 0.998 |
| mttB | 376 | 126 | CCC->UCC | Pro->Ser | TGTTCCTGTTCCTAACTCCT | C | CCCGGGTAGTTCCCAATGTT | 0.99 |
| mttB | 379 | 127 | CGG->UGG | Arg->Trp | TCCTGTTCCTAACTCCTCCC | C | GGGTAGTTCCCAATGTTTGG | 0.995 |
| mttB | 407 | 136 | CCA->CUA | Pro->Leu | TCCCAATGTTTGGCACTTTC | C | ATACTTCGTGGGTGCAACAT | 0.999 |
| mttB | 472 | 158 | CAU->UAU | His->Tyr | TACAACCTAAGATCTATGAC | C | ATATTATGTTAACTGTTCGT | 0.997 |
| mttB | 497 | 166 | UCG->UUG | Ser->Leu | TATGTTAACTGTTCGTATTT | C | GTTCATTCCATCGGTATGCT | 0.994 |
| mttB | 505 | 169 | CCA->UCA | Pro->Ser | CTGTTCGTATTTCGTTCATT | C | CATCGGTATGCTCCCAGGTA | 0.995 |
| mttB | 541 | 181 | CGU->UGU | Arg->Cys | AGGTACCTGTAATTGTGATC | C | GTTTGCCAGAACGAAGGGGT | 0.986 |
| mttB | 548 | 183 | CCA->CUA | Pro->Leu | TGTAATTGTGATCCGTTTGC | C | AGAACGAAGGGGTCTTTCTG | 1 |
| mttB | 610 | 204 | CCG->UCG | Pro->Ser | GTCGTTTTTTGATGGTTTTT | C | CGCTTCTCACAGCTGCTCTT | 0.997 |
| mttB | 616 | 206 | CUC->UUC | Leu->Phe | TTTTGATGGTTTTTCCGCTT | C | TCACAGCTGCTCTTTCCACA | 0.971 |
| mttB | 660 | 220 | AUC->AUU | Ile->Ile | CCGGATATCTGGTGCCAAAT | C | GTCGCCCTTTTCCTTATTTC | 0.951 |
| mttB | 667 | 223 | CUU->UUU | Leu->Phe | TCTGGTGCCAAATCGTCGCC | C | TTTTCCTTATTTCTTCGATA | 0.972 |
| mttB | 683 | 228 | UCG->UUG | Ser->Leu | CGCCCTTTTCCTTATTTCTT | C | GATAATAGAGTTGGCTATCT | 0.995 |
| mttB | 713 | 238 | UCG->UUG | Ser->Leu | GTTGGCTATCTTTGTGGCAT | C | GATTGTACAAGTTCGTGAAG | 0.999 |
| nad1 | 2 | 1 | ACG->AUG | Thr->Met | AGTGAATAGAAAATCGAAAA | C | GTACATAGCTGTTCCAGCTG | 0.992 |
| nad1 | 215 | 72 | UCC->UUC | Ser->Phe | ACCAAGTAGTGCTAATTTCT | C | CCTTTTTAGAATGGCTCCAG | 1 |
| nad1 | 308 | 103 | UCG->UUG | Ser->Leu | TGGTATGGTATTGTCAGATT | C | GAACATAGGGCTACTTTATT | 0.999 |
| nad1 | 436 | 146 | CCU->UCU | Pro->Ser | GATCTGCAGCTCAAATGGTC | C | CTTATGAAGTCTCTATTGGT | 0.998 |
| nad1 | 490 | 164 | CCC->UCU | Pro->Ser | CTGTACTAATATGTGTAGGT | C | CCCGTAATTCGAGTGAGATT | 0.997 |
| nad1 | 492 | 164 | CCC->UCU | Pro->Ser | GTACTAATATGTGTAGGTCC | C | CGTAATTCGAGTGAGATTGT | 0.989 |
| nad1 | 493 | 165 | CGU->UGU | Arg->Cys | TACTAATATGTGTAGGTCCC | C | GTAATTCGAGTGAGATTGTC | 0.995 |
| nad1 | 500 | 167 | UCG->UUG | Ser->Leu | ATGTGTAGGTCCCCGTAATT | C | GAGTGAGATTGTCATGGCGC | 1 |
| nad1 | 536 | 179 | UCC->UUU | Ser->Phe | GGCGCAAAAGCAGATATGGT | C | CGGTATTCCCTTGTTCCCTG | 0.997 |
| nad1 | 537 | 179 | UCC->UUU | Ser->Phe | GCGCAAAAGCAGATATGGTC | C | GGTATTCCCTTGTTCCCTGT | 0.993 |
| nad1 | 573 | 191 | UUC->UUU | Phe->Phe | CCTGTATTGGTTATGTTCTT | C | ATTTCTCGTCTAGCAGAAAC | 1 |
| nad1 | 580 | 194 | CGU->UGU | Arg->Cys | TGGTTATGTTCTTCATTTCT | C | GTCTAGCAGAAACTAATCGG | 0.997 |
| nad1 | 635 | 212 | UCA->UUA | Ser->Leu | CCCAGAAGCGGAAGCTGAAT | C | AGTTGCAGGCTATAATGTGG | 0.997 |
| nad1 | 674 | 225 | UCU->UUU | Ser->Phe | GGAATATTCTTCAATGGGGT | C | TGCTCTTTTTTTTTTGGGAG | 1 |
| nad1 | 725 | 242 | CCA->CUA | Pro->Leu | TATGATCTTAATGAGTGGTC | C | ATGCACATCGCTCTCTCCAG | 0.995 |
| nad1 | 734 | 245 | UCG->UUG | Ser->Leu | AATGAGTGGTCCATGCACAT | C | GCTCTCTCCAGGAGGTTGGC | 0.997 |
| nad1 | 740 | 247 | UCU->UUU | Ser->Phe | TGGTCCATGCACATCGCTCT | C | TCCAGGAGGTTGGCCGCCTA | 0.999 |
| nad1 | 743 | 248 | CCA->CUA | Pro->Leu | TCCATGCACATCGCTCTCTC | C | AGGAGGTTGGCCGCCTATCC | 0.999 |
| nad1 | 755 | 252 | CCG->CUG | Pro->Leu | GCTCTCTCCAGGAGGTTGGC | C | GCCTATCCTAGATCTTCCCA | 0.995 |
| nad1 | 823 | 275 | CUC->UUC | Leu->Phe | GGTTTAGTATCAAGGTGATT | C | TCTTTCTGTTCCTATATATA | 0.99 |
| nad1 | 898 | 300 | CGG->UGG | Arg->Trp | ATCAATTAATGGGACTTGGC | C | GGAAAGTGTTCTTGCCTCTA | 0.999 |
| nad1 | 928 | 310 | CGG->UGG | Arg->Trp | TCTTGCCTCTATCATTAGCT | C | GGGTAGTCCCCGTTTCTGGT | 0.999 |
| nad1 | 937 | 313 | CCC->UCC | Pro->Ser | TATCATTAGCTCGGGTAGTC | C | CCGTTTCTGGTGTTTCAGTC | 0.993 |
| nad1 | 953 | 318 | UCA->UUA | Ser->Leu | AGTCCCCGTTTCTGGTGTTT | C | AGTCACCTTTCAATGGCTCC | 0.998 |
| nad2 | 26 | 9 | UCC->UUC | Ser->Phe | CAATCTTTTTTTAGCGGTTT | C | CCCAGAGATCTTTATCATTA | 0.996 |
| nad2 | 223 | 75 | CUU->UUU | Leu->Phe | CCCATTTATTCTGGAATAAT | C | TTTTTAGGAGGGACAATTTT | 0.997 |
| nad2 | 308 | 103 | UCU->UUU | Ser->Phe | CATTTCGATGTGTTTTGATT | C | TTCCGAACAAGAGAGGTTTG | 0.995 |
| nad2 | 311 | 104 | UCC->UUC | Ser->Phe | TTCGATGTGTTTTGATTCTT | C | CGAACAAGAGAGGTTTGATG | 0.998 |
| nad2 | 356 | 119 | CCA->CUA | Pro->Leu | TGAATTCATTGTATTAATTC | C | ACTTCCTACTCGCAGTATGC | 0.995 |
| nad2 | 361 | 121 | CCU->UCU | Pro->Ser | TCATTGTATTAATTCCACTT | C | CTACTCGCAGTATGCTCTTT | 0.999 |
| nad2 | 367 | 123 | CGC->UGC | Arg->Cys | TATTAATTCCACTTCCTACT | C | GCAGTATGCTCTTTATGATC | 0.989 |
| nad2 | 394 | 132 | CAU->UAU | His->Tyr | TGCTCTTTATGATCTCGGCT | C | ATGATTCAATTGCCATGTAT | 0.997 |
| nad2 | 401 | 134 | UCA->UUA | Ser->Leu | TATGATCTCGGCTCATGATT | C | AATTGCCATGTATTTAGCTA | 0.995 |
| nad2 | 428 | 143 | CCU->CUU | Pro->Leu | CATGTATTTAGCTATTGAAC | C | TCAAAGTTTATGTTTTTATG | 1 |
| nad2 | 497 | 166 | UCG->UUG | Ser->Leu | ATTTTCCACGGAAGCCGGCT | C | GAAATATTTGATCTTAGGTG | 0.999 |
| nad2 | 523 | 175 | CCC->UCC | Pro->Ser | ATTTGATCTTAGGTGCATTT | C | CCTCTGGAATATTACTGTTT | 0.992 |
| nad2 | 662 | 221 | UCU->UUU | Ser->Phe | TATTTTTATGGGGATTCTAT | C | TATCGCTGTAGGATTCCTAT | 0.999 |
| nad2 | 788 | 263 | UCU->UUU | Ser->Phe | TGCGCCTAAAATATCTATTT | C | TGCTAATATTTCACGTGTTT | 0.999 |
| nad2 | 800 | 267 | UCA->UUA | Ser->Leu | ATCTATTTCTGCTAATATTT | C | ACGTGTTTCTATTTATGGTT | 0.966 |
| nad2 | 809 | 270 | UCU->UUU | Ser->Phe | TGCTAATATTTCACGTGTTT | C | TATTTATGGTTCCTATGGAG | 0.998 |
| nad2 | 920 | 307 | CCU->CUU | Pro->Leu | CCAAACGAAAGTCAAAAGAC | C | TCTAGCTCATAGTTCAATTG | 0.999 |
| nad2 | 928 | 310 | CAU->UAU | His->Tyr | AAGTCAAAAGACCTCTAGCT | C | ATAGTTCAATTGGCCATGTA | 0.998 |
| nad2 | 958 | 320 | CGU->UGU | Arg->Cys | TTGGCCATGTAGGTTATATT | C | GTACTGGTTTCTCATGTGGA | 1 |
| nad2 | 962 | 321 | ACU->AUU | Thr->Ile | CCATGTAGGTTATATTCGTA | C | TGGTTTCTCATGTGGAACCA | 0.995 |
| nad2 | 1028 | 343 | UCA->UUA | Ser->Leu | TGGTATCTTTATTTATGCAT | C | AATGACGATAGATGCATTCG | 0.999 |
| nad2 | 1058 | 353 | UCA->UUA | Ser->Leu | AGATGCATTCGCCATAGTTT | C | AGCATTACGGCAAACCCGTG | 0.997 |
| nad2 | 1127 | 376 | UCG->UUG | Ser->Leu | AGCCAAAACTAATCCTATTT | C | GGCTATTACCTTCTCCATTA | 0.994 |
| nad2 | 1246 | 416 | CCA->UUA | Pro->Leu | GTGGGGCTTACTTTCTAGCC | C | CAGTGGGAGTAGTGACTAGC | 0.999 |
| nad2 | 1247 | 416 | CCA->UUA | Pro->Leu | TGGGGCTTACTTTCTAGCCC | C | AGTGGGAGTAGTGACTAGCG | 0.988 |
| nad2 | 1276 | 426 | CGU->UGU | Arg->Cys | TAGTGACTAGCGTTATAGGT | C | GTTTTTATTATATACGCTTA | 1 |
| nad2 | 1298 | 433 | GCG->GUG | Ala->Val | TTTTTATTATATACGCTTAG | C | GAAAAGAATGTTTTTTGATA | 1 |
| nad2 | 1400 | 467 | UCA->UUA | Ser->Leu | GACTTCCTCTTTCATTACTT | C | ATCCTTTCCATATCCCTCTC | 0.99 |
| nad2 | 1403 | 468 | UCC->UUC | Ser->Phe | TTCCTCTTTCATTACTTCAT | C | CTTTCCATATCCCTCTCCCT | 0.99 |
| nad2 | 1408 | 470 | CCA->UUA | Pro->Leu | CTTTCATTACTTCATCCTTT | C | CATATCCCTCTCCCTTGTTC | 0.993 |
| nad2 | 1409 | 470 | CCA->UUA | Pro->Leu | TTTCATTACTTCATCCTTTC | C | ATATCCCTCTCCCTTGTTCT | 0.99 |
| nad2 | 1416 | 472 | CCC->CCU | Pro->Pro | ACTTCATCCTTTCCATATCC | C | TCTCCCTTGTTCTCAGTTAC | 0.955 |
| nad3 | 5 | 2 | UCA->UUA | Ser->Leu | CAAGTGGGCTGTGGTGATGT | C | AGAATTTGCACCTATTTGTA | 0.998 |
| nad3 | 61 | 21 | CCA->UUA | Pro->Leu | GTCTGCTAGTTTCTTTGATC | C | CACTCGGTGTTCCTTTTCCA | 0.976 |
| nad3 | 62 | 21 | CCA->UUA | Pro->Leu | TCTGCTAGTTTCTTTGATCC | C | ACTCGGTGTTCCTTTTCCAT | 0.999 |
| nad3 | 80 | 27 | CCA->CUA | Pro->Leu | CCCACTCGGTGTTCCTTTTC | C | ATTTGCTTCCAATAGTTCAA | 1 |
| nad3 | 146 | 49 | UCC->UUC | Ser->Phe | CGAATGTGGTTTCGATCCTT | C | CGGTGATGCCAGAAGTCGTT | 1 |
| nad3 | 208 | 70 | CUU->UUU | Leu->Phe | TTTCAATTTTATTTATTATC | C | TTGATCCTGAAGTAACCTTT | 0.957 |
| nad3 | 215 | 72 | CCU->CUU | Pro->Leu | TTTATTTATTATCCTTGATC | C | TGAAGTAACCTTTTCCTTTC | 0.966 |
| nad3 | 230 | 77 | UCC->UUC | Ser->Phe | TGATCCTGAAGTAACCTTTT | C | CTTTCCTTGGGCAGTACCTC | 0.999 |
| nad3 | 247 | 83 | CCU->UCU | Pro->Ser | TTTCCTTTCCTTGGGCAGTA | C | CTCTCAACAAGATTGATCTG | 0.996 |
| nad3 | 275 | 92 | UCU->UUU | Ser->Phe | CAAGATTGATCTGTTTGGAT | C | TTGGTCCATGATGGCCTTTT | 0.995 |
| nad3 | 317 | 106 | UCU->UUU | Ser->Phe | ATTGATTTTGACGATTGGAT | C | TCTCTATGAATGGAAAAGGG | 1 |
| nad3 | 344 | 115 | UCG->UUG | Ser->Leu | TGAATGGAAAAGGGGTGCTT | C | GGATCGGGAGTAATCACTAG | 0.998 |
| nad3 | 349 | 117 | CGG->UGG | Arg->Trp | GGAAAAGGGGTGCTTCGGAT | C | GGGAGTAATCACTAGTGATA | 1 |
| nad4 | 29 | 10 | UCU->UUU | Ser->Phe | ACATTTCTGTGAATGCTATT | C | TGATCTAAGTGGTCTTATTC | 1 |
| nad4 | 74 | 25 | ACU->AUU | Thr->Ile | TCCCGTGCTAGGAAGCATTA | C | TCCTCTTTTCATTCCAAATT | 0.997 |
| nad4 | 77 | 26 | CCU->CUU | Pro->Leu | CGTGCTAGGAAGCATTACTC | C | TCTTTTCATTCCAAATTCAA | 0.999 |
| nad4 | 107 | 36 | CCG->CUG | Pro->Leu | TCCAAATTCAAGAATACGAC | C | GATACGATTGATTGGTCTGT | 0.999 |
| nad4 | 154 | 52 | CCC->UCC | Pro->Ser | CTCTTATTACTTTTTTGTAT | C | CCCCTGTTCCTCGGATACAA | 0.999 |
| nad4 | 158 | 53 | CCU->CUU | Pro->Leu | TATTACTTTTTTGTATCCCC | C | TGTTCCTCGGATACAATTCG | 0.997 |
| nad4 | 164 | 55 | CCU->CUU | Pro->Leu | TTTTTTGTATCCCCCTGTTC | C | TCGGATACAATTCGATCCTT | 0.994 |
| nad4 | 166 | 56 | CGG->UGG | Arg->Trp | TTTTGTATCCCCCTGTTCCT | C | GGATACAATTCGATCCTTCT | 1 |
| nad4 | 197 | 66 | UCU->UUU | Ser->Phe | CGATCCTTCTACGGCCAAAT | C | TCAATTTGTGGAAAGCCTTC | 0.998 |
| nad4 | 317 | 106 | UCA->UUA | Ser->Leu | TCTGATCCCTATTTGCATTT | C | AGTGGGTTGGTCTGGTATGA | 0.999 |
| nad4 | 362 | 121 | ACA->AUA | Thr->Ile | TTATGGGAAAGAGTATATTA | C | AGCATCTCTAATTCGTGAAT | 0.999 |
| nad4 | 368 | 123 | UCU->UUU | Ser->Phe | GAAAGAGTATATTACAGCAT | C | TCTAATTCGTGAATTTCTAA | 0.996 |
| nad4 | 376 | 126 | CGU->UGU | Arg->Cys | ATATTACAGCATCTCTAATT | C | GTGAATTTCTAATGATCGCC | 0.995 |
| nad4 | 416 | 139 | CCU->CUU | Pro->Leu | CGTGTTCTGCATGCTGGATC | C | TTTACTATTCTATGTTCTTC | 0.977 |
| nad4 | 433 | 145 | CUU->UUU | Leu->Phe | ATCCTTTACTATTCTATGTT | C | TTCCCGAAAGCGTGCTAATC | 0.993 |
| nad4 | 436 | 146 | CCC->UUC | Pro->Phe | CTTTACTATTCTATGTTCTT | C | CCGAAAGCGTGCTAATCCCT | 0.993 |
| nad4 | 437 | 146 | CCC->UUC | Pro->Phe | TTTACTATTCTATGTTCTTC | C | CGAAAGCGTGCTAATCCCTA | 0.995 |
| nad4 | 608 | 203 | UCA->UUA | Ser->Leu | AACCACCGATTTACAAATTT | C | ATTAACCACAGAATTTAGTG | 0.998 |
| nad4 | 659 | 220 | UCU->UUU | Ser->Phe | AATCTTTCTATGGATTGCTT | C | TTTCGCCTCTTTCGCCGTCA | 0.999 |
| nad4 | 767 | 256 | CCA->CUA | Pro->Leu | CGTCATCTTGGCAGGAATTC | C | ATTAAAATTGGGAACCCACG | 1 |
| nad4 | 784 | 262 | CAC->UAC | His->Tyr | TTCCATTAAAATTGGGAACC | C | ACGGCTTTTTAAGATTTTCA | 0.99 |
| nad4 | 836 | 279 | UCC->UUC | Ser->Phe | TCCCGAAGCGACACTTTGTT | C | CACTCCTTTCATTTATACTC | 0.995 |
| nad4 | 856 | 286 | CCA->UUA | Pro->Leu | CCACTCCTTTCATTTATACT | C | CAAGCGCGATTGCTATAATA | 0.979 |
| nad4 | 857 | 286 | CCA->UUA | Pro->Leu | CACTCCTTTCATTTATACTC | C | AAGCGCGATTGCTATAATAT | 1 |
| nad4 | 896 | 299 | UCA->UUA | Ser->Leu | ATATACTTCCTTGACCACTT | C | AAGACAGATCGATCTTAAGA | 1 |
| nad4 | 977 | 326 | CCG->CUG | Pro->Leu | GACTATTGGTATGTTTAGTC | C | GAACATACAGGGAATTGGAG | 1 |
| nad4 | 1006 | 336 | CCA->UUA | Pro->Leu | AGGGAATTGGAGGTAGCATT | C | CACCGATGTTAAGTCATGGA | 0.976 |
| nad4 | 1007 | 336 | CCA->UUA | Pro->Leu | GGGAATTGGAGGTAGCATTC | C | ACCGATGTTAAGTCATGGAC | 1 |
| nad4 | 1010 | 337 | CCG->CUG | Pro->Leu | AATTGGAGGTAGCATTCCAC | C | GATGTTAAGTCATGGACTGG | 0.999 |
| nad4 | 1033 | 345 | CCU->UCU | Pro->Ser | TGTTAAGTCATGGACTGGTT | C | CTTCAGCCCTTTTTCTATGT | 0.999 |
| nad4 | 1101 | 367 | UAC->UAU | Tyr->Tyr | ACTCGACTTGTTAGATATTA | C | GGAGGTTCAGTGAGCACCAT | 0.906 |
| nad4 | 1109 | 370 | UCA->UUA | Ser->Leu | TGTTAGATATTACGGAGGTT | C | AGTGAGCACCATGCCGAATC | 0.999 |
| nad4 | 1129 | 377 | CUC->UUC | Leu->Phe | CAGTGAGCACCATGCCGAAT | C | TCCCTACCATTTCCTTCTCT | 0.995 |
| nad4 | 1132 | 378 | CCU->UCU | Pro->Ser | TGAGCACCATGCCGAATCTC | C | CTACCATTTCCTTCTCTTCC | 0.998 |
| nad4 | 1142 | 381 | UCC->UUC | Ser->Phe | GCCGAATCTCCCTACCATTT | C | CTTCTCTTCCACTTTGGCCA | 0.99 |
| nad4 | 1148 | 383 | UCU->UUU | Ser->Phe | TCTCCCTACCATTTCCTTCT | C | TTCCACTTTGGCCAATATGA | 0.998 |
| nad4 | 1151 | 384 | UCC->UUC | Ser->Phe | CCCTACCATTTCCTTCTCTT | C | CACTTTGGCCAATATGAGTT | 0.992 |
| nad4 | 1172 | 391 | UCA->UUA | Ser->Leu | CACTTTGGCCAATATGAGTT | C | ACCTGGTACTAGCAGCTTTA | 1 |
| nad4 | 1205 | 402 | CCC->CUU | Pro->Leu | CAGCTTTATCGGGGAATTTC | C | CATCTCAGTAGGAGCTTTCC | 1 |
| nad4 | 1206 | 402 | CCC->CUU | Pro->Leu | AGCTTTATCGGGGAATTTCC | C | ATCTCAGTAGGAGCTTTCCA | 0.96 |
| nad4 | 1211 | 404 | UCA->UUA | Ser->Leu | TATCGGGGAATTTCCCATCT | C | AGTAGGAGCTTTCCAAAGAA | 0.997 |
| nad4 | 1355 | 452 | CCA->CUA | Pro->Leu | CCTCCATAAATTCTCCGATC | C | AAATGGCAGAGAAGTTTCCA | 0.995 |
| nad4 | 1373 | 458 | UCC->UUC | Ser->Phe | TCCAAATGGCAGAGAAGTTT | C | CATATTTATACCTTTTCTTG | 0.999 |
| nad4 | 1405 | 469 | CGG->UGG | Arg->Trp | CTTTTCTTGTTGGAGTTGTT | C | GGATGGGTGTTCACCCCAAA | 0.999 |
| nad4 | 1417 | 473 | CAC->UAC | His->Tyr | GAGTTGTTCGGATGGGTGTT | C | ACCCCAAAGTGTTCCCGGAC | 0.999 |
| nad4 | 1433 | 478 | CCG->CUG | Pro->Leu | TGTTCACCCCAAAGTGTTCC | C | GGACCGCATGCATACATCCG | 0.999 |
| nad4 | 1438 | 480 | CGC->UGC | Arg->Cys | ACCCCAAAGTGTTCCCGGAC | C | GCATGCATACATCCGTAAGT | 0.991 |
| nad5 | 155 | 52 | CCG->CUG | Pro->Leu | TGCTTTTTATGAAGTCGCAC | C | GGGAGCTAGTGCTTGCTATC | 1 |
| nad5 | 242 | 81 | CCG->CUG | Pro->Leu | GGGCTTCTTGTTCGATAGCC | C | GACCGTAGTGATGTTAATTG | 1 |
| nad5 | 272 | 91 | UCC->UUC | Ser->Phe | GATGTTAATTGTGGTTACAT | C | CATAAGTAGCTTGGTCCATC | 0.999 |
| nad5 | 374 | 125 | CCA->CUA | Pro->Leu | CATTTTTACTTTTTTTATGC | C | AATGTTGGTGACTGGAGATA | 0.995 |
| nad5 | 398 | 133 | UCU->UUU | Ser->Phe | GTTGGTGACTGGAGATAACT | C | TCTTCAATTATTCCTGGGAT | 1 |
| nad5 | 494 | 165 | ACA->AUA | Thr->Ile | TCAGGCAGATAAAGCAGCTA | C | AAAAGCTATGCCTGTCAATC | 1 |
| nad5 | 506 | 169 | CCU->CUU | Pro->Leu | AGCAGCTACAAAAGCTATGC | C | TGTCAATCGAGTAGGGGATT | 0.932 |
| nad5 | 548 | 183 | UCG->UUG | Ser->Leu | TGGATTAGCTCCTGGGATTT | C | GGGCCGTTTTACTCTCTTTC | 0.998 |
| nad5 | 553 | 185 | CGU->UGU | Arg->Cys | TAGCTCCTGGGATTTCGGGC | C | GTTTTACTCTCTTTCAAACA | 0.991 |
| nad5 | 598 | 200 | CGU->UGU | Arg->Cys | ACTTTTCAACCATTTTTGCT | C | GTGCTAGTGCCCCCAGAAAT | 0.999 |
| nad5 | 608 | 203 | GCC->GUC | Ala->Val | CATTTTTGCTCGTGCTAGTG | C | CCCCAGAAATTCTTGGATTT | 0.993 |
| nad5 | 629 | 210 | UCU->UUU | Ser->Phe | CCCCAGAAATTCTTGGATTT | C | TTGCAATATGAGATTGAATG | 0.999 |
| nad5 | 713 | 238 | UCG->UUG | Ser->Leu | GAAATCTGCACAGATAGGAT | C | GCATACTTGGTCACCCGATG | 0.999 |
| nad5 | 725 | 242 | UCA->UUA | Ser->Leu | GATAGGATCGCATACTTGGT | C | ACCCGATGCTATGGAGGGTC | 1 |
| nad5 | 764 | 255 | UCG->UUG | Ser->Leu | TCCCACTCCAGTATCCGCTT | C | GATTCATGCAGCTACTATGG | 0.995 |
| nad5 | 835 | 279 | CCA->UCA | Pro->Ser | GCTCCCCTTTATTTGAATAC | C | CACCTACGGCTTTGATTGTT | 1 |
| nad5 | 863 | 288 | UCU->UUU | Ser->Phe | GGCTTTGATTGTTATTACTT | C | TGCAGGAGCTACGACGTCAT | 0.999 |
| nad5 | 875 | 292 | ACG->AUG | Thr->Met | TATTACTTCTGCAGGAGCTA | C | GACGTCATTCCTTGCGGCAA | 1 |
| nad5 | 1310 | 437 | UCA->UUA | Ser->Leu | TTCTTATTACTCTTTTCGTT | C | ACTTTTTCTAACATTTCTAG | 0.999 |
| nad5 | 1400 | 467 | UCA->UUA | Ser->Leu | CATTCCTATGGCCATTCCTT | C | AATACTTCTGGCTTTCGGGA | 0.999 |
| nad5 | 1490 | 497 | CCC->CUC | Pro->Leu | CCATTTTTGGGCCAATTCCC | C | CTTCGTACTACCAAAAAATG | 0.997 |
| nad5 | 1550 | 517 | ACC->AUC | Thr->Ile | GTTTGCTGCTCCAACCATTA | C | CAAACTAATACCTATTCCAT | 0.976 |
| nad5 | 1568 | 523 | CCA->CUA | Pro->Leu | TACCAAACTAATACCTATTC | C | ATTTAGTACTTCAGGTGCTT | 0.999 |
| nad5 | 1580 | 527 | UCA->UUA | Ser->Leu | ACCTATTCCATTTAGTACTT | C | AGGTGCTTCTGTTGCGTATA | 0.995 |
| nad5 | 1589 | 530 | UCU->UUU | Ser->Phe | ATTTAGTACTTCAGGTGCTT | C | TGTTGCGTATAATGTAAATC | 0.967 |
| nad5 | 1610 | 537 | CCC->CUC | Pro->Leu | TGTTGCGTATAATGTAAATC | C | CGTAGCGGATCAATTCCAAC | 0.999 |
| nad5 | 1895 | 632 | UCA->UUA | Ser->Leu | CTTTGCAATGTTACTTGGTT | C | AACTCTATTTGTGACCCTTT | 0.998 |
| nad5 | 1916 | 639 | UCU->UUU | Ser->Phe | AACTCTATTTGTGACCCTTT | C | TCGTATGTGGGACTCTCTAT | 0.997 |
| nad5 | 1918 | 640 | CGU->UGU | Arg->Cys | CTCTATTTGTGACCCTTTCT | C | GTATGTGGGACTCTCTATCT | 0.981 |
| nad5 | 1958 | 653 | UCG->UUG | Ser->Leu | TTCTTGGGTAGATAATCGAT | C | GTCTTTCATTTTGATAGTGA | 0.972 |
| nad6 | 26 | 9 | CCU->CUU | Pro->Leu | ACTTTCTGTTTTGTCGAGCC | C | TGCTTTGGTCTCTGGTTTGA | 0.999 |
| nad6 | 53 | 18 | GCA->GUA | Ala->Val | GGTCTCTGGTTTGATGGTTG | C | ACGTGCTAAAAATCCGGTAC | 1 |
| nad6 | 88 | 30 | CCC->UUC | Pro->Phe | CGGTACATTCCGTTTTGTTT | C | CCATTTCAGTCTTTCGCGAC | 0.995 |
| nad6 | 89 | 30 | CCC->UUC | Pro->Phe | GGTACATTCCGTTTTGTTTC | C | CATTTCAGTCTTTCGCGACA | 0.988 |
| nad6 | 95 | 32 | UCA->UUA | Ser->Leu | TTCCGTTTTGTTTCCCATTT | C | AGTCTTTCGCGACACTTCAG | 0.987 |
| nad6 | 103 | 35 | CGC->UGC | Arg->Cys | TGTTTCCCATTTCAGTCTTT | C | GCGACACTTCAGGTTTACTT | 0.978 |
| nad6 | 161 | 54 | CCA->CUA | Pro->Leu | CTTCTTCGCTATGATCTTCC | C | AGTAGTTCATATAGGAGCTA | 1 |
| nad6 | 169 | 57 | CAU->UAU | His->Tyr | CTATGATCTTCCCAGTAGTT | C | ATATAGGAGCTATAGCCGTC | 1 |
| nad6 | 446 | 149 | UCC->UUC | Ser->Phe | TTTACTTTATACCTACTATT | C | CGTCTGGTTTTTGGTTCCTA | 0.999 |
| nad6 | 463 | 155 | CCU->UCU | Pro->Ser | ATTCCGTCTGGTTTTTGGTT | C | CTAGTCTTATTTTATTAGTA | 0.992 |
| nad7 | 38 | 13 | UCG->UUG | Ser->Leu | GCAAATCAAAAATTTCACTT | C | GAATTCCGGACCTCAACATC | 0.998 |
| nad7 | 44 | 15 | UCC->UUU | Ser->Phe | CAAAAATTTCACTTCGAATT | C | CGGACCTCAACATCCTGCTG | 0.996 |
| nad7 | 45 | 15 | UCC->UUU | Ser->Phe | AAAAATTTCACTTCGAATTC | C | GGACCTCAACATCCTGCTGC | 0.998 |
| nad7 | 77 | 26 | UCA->UUA | Ser->Leu | TCCTGCTGCTCATGGTGTTT | C | ACGATCAGTATTGGAAATGA | 0.991 |
| nad7 | 83 | 28 | UCA->UUA | Ser->Leu | TGCTCATGGTGTTTCACGAT | C | AGTATTGGAAATGAACGGAG | 1 |
| nad7 | 137 | 46 | UCA->UUA | Ser->Leu | TGCGGAACCACATATTGGAT | C | ACTCCATAGAGGGACTGAGA | 0.999 |
| nad7 | 200 | 67 | UCU->UUU | Ser->Phe | TCTTCAAGCTTTACCTTATT | C | TGATCGTTCAGACTATGTTT | 0.995 |
| nad7 | 209 | 70 | UCA->UUA | Ser->Leu | TTTACCTTATTCTGATCGTT | C | AGACTATGTTTCTACGATGG | 0.989 |
| nad7 | 224 | 75 | ACG->AUG | Thr->Met | TCGTTCAGACTATGTTTCTA | C | GATGGCCCAAGAACACGCTC | 1 |
| nad7 | 244 | 82 | CAU->UAU | His->Tyr | CGATGGCCCAAGAACACGCT | C | ATTCTTCAGCCGTAGAGAGA | 0.999 |
| nad7 | 251 | 84 | UCA->UUA | Ser->Leu | CCAAGAACACGCTCATTCTT | C | AGCCGTAGAGAGACTTTTGA | 0.993 |
| nad7 | 316 | 106 | CGU->UGU | Arg->Cys | AATATATACGAGTGTTATTC | C | GTGAAATAACTCGAATTTCA | 0.997 |
| nad7 | 335 | 112 | UCA->UUA | Ser->Leu | CCGTGAAATAACTCGAATTT | C | AAATCATTCACTTGCTTTAA | 0.999 |
| nad7 | 344 | 115 | UCA->UUA | Ser->Leu | AACTCGAATTTCAAATCATT | C | ACTTGCTTTAACTACTCATG | 0.999 |
| nad7 | 383 | 128 | UCA->UUA | Ser->Leu | TGCTATGGATGTGGGAGCAT | C | AACTCCGTTCCTGTGGGCTT | 1 |
| nad7 | 404 | 135 | UCU->UUU | Ser->Phe | AACTCCGTTCCTGTGGGCTT | C | TGAGGAGCGGGAGAAATTGT | 1 |
| nad7 | 533 | 178 | UCC->UUC | Ser->Phe | ATGTCGAGATATTGATTCCT | C | CACACAACAATTTGCTTCTC | 0.998 |
| nad7 | 578 | 193 | UCA->UUA | Ser->Leu | CGACGAATTAGAAGAGATGT | C | AACCGGCAACCGTATCTGGA | 1 |
| nad7 | 679 | 227 | CCA->UCA | Pro->Ser | GTGGTGTAATGTTAAGAGGT | C | CAGGGGTATGCTGGGATTCG | 0.987 |
| nad7 | 698 | 233 | UCG->UUG | Ser->Leu | TCCAGGGGTATGCTGGGATT | C | GCGAAGAGCAGCACCTTACG | 1 |
| nad7 | 724 | 242 | CAU->UAU | His->Tyr | GAGCAGCACCTTACGATGTT | C | ATGACCAATTGGATCCTGAC | 1 |
| nad7 | 739 | 247 | CCU->UUU | Pro->Phe | ATGTTCATGACCAATTGGAT | C | CTGACGTACCAGTAGGTACC | 0.999 |
| nad7 | 740 | 247 | CCU->UUU | Pro->Phe | TGTTCATGACCAATTGGATC | C | TGACGTACCAGTAGGTACCA | 0.999 |
| nad7 | 769 | 257 | CGC->UGC | Arg->Cys | CAGTAGGTACCAGAGGAGAT | C | GCTATGATCGTTACTGTATC | 1 |
| nad7 | 836 | 279 | CCU->CUU | Pro->Leu | TCGGATCATTGTGCAATGTC | C | TAATCAAATGCCTAGTGGCA | 0.999 |
| nad7 | 926 | 309 | UCA->UUA | Ser->Leu | GAAACTATCCATGGAATCCT | C | AATTCACCATTTCGAACCTT | 0.999 |
| nad7 | 944 | 315 | CCU->CUU | Pro->Leu | CTCAATTCACCATTTCGAAC | C | TTATACAGAAGGTTTTTCCG | 0.999 |
| nad7 | 973 | 325 | CCU->UCU | Pro->Ser | AAGGTTTTTCCGTACCAGCT | C | CTTCTACCTATACCGCAGTT | 0.999 |
| nad7 | 1050 | 350 | CCC->CCU | Pro->Pro | AGTAATGGAAGCAATCGTCC | C | TACCGTCGTAAAATAAGAGC | 0.966 |
| nad7 | 1057 | 353 | CGU->UGU | Arg->Cys | GAAGCAATCGTCCCTACCGT | C | GTAAAATAAGAGCACCTGGC | 0.998 |
| nad7 | 1079 | 360 | UCU->UUU | Ser->Phe | TAAAATAAGAGCACCTGGCT | C | TGCCCATTCACAAGGACTCG | 0.999 |
| nad7 | 1088 | 363 | UCA->UUA | Ser->Leu | AGCACCTGGCTCTGCCCATT | C | ACAAGGACTCGATTCTATGT | 0.996 |
| nad7 | 1103 | 368 | UCU->UUU | Ser->Phe | CCATTCACAAGGACTCGATT | C | TATGTCCAAACATCACATGC | 0.999 |
| nad7 | 1124 | 375 | CCA->CUA | Pro->Leu | TATGTCCAAACATCACATGC | C | AGCAGATGTGGTCACCATCA | 1 |
| nad7 | 1137 | 379 | GUC->GUU | Val->Val | CACATGCCAGCAGATGTGGT | C | ACCATCATAGGTACTCAAGA | 0.941 |
| nad7 | 1166 | 389 | UCU->UUU | Ser->Phe | AGGTACTCAAGATATTGTGT | C | TGGAGAGGTGGATAGATAGG | 1 |
| nad9 | 92 | 31 | UCU->UUU | Ser->Phe | ATCAGAACATGGGAATAGAT | C | TGATACCAATACGGACTACC | 0.963 |
| nad9 | 113 | 38 | CCA->CUA | Pro->Leu | TGATACCAATACGGACTACC | C | ATTTCAATTGTTGTGCTTTC | 1 |
| nad9 | 167 | 56 | UCG->UUG | Ser->Leu | CTATACAAGGGTTCAAGTTT | C | GATCGATATTTGCGGAGTGG | 0.998 |
| nad9 | 298 | 100 | CCG->UCG | Pro->Ser | ACGAAGTAACACGAATATCT | C | CGGTAGTCAGTCTATTTCCA | 0.996 |
| nad9 | 328 | 110 | CGG->UGG | Arg->Trp | GTCTATTTCCATCAGCCGGC | C | GGTGGGAGCGAGAAGTTTGG | 1 |
| nad9 | 368 | 123 | UCC->UUC | Ser->Phe | GGATATGTTTGGTGTTTCTT | C | CATCAATCATCCGGATCTAC | 1 |
| nad9 | 398 | 133 | UCA->UUA | Ser->Leu | TCCGGATCTACGCCGTATAT | C | AACAGATTATGGTTTCGAGG | 0.998 |
| nad9 | 439 | 147 | CUU->UUU | Leu->Phe | GTCATCCATTACGAAAAGAC | C | TTCCTCTGAGTGGATATGTG | 0.998 |
| rpl16 | 102 | 34 | GGC->GGU | Gly->Gly | CTTGGTTTTGGAAGATATGG | C | ACTAAAAGTTGTAGAGCTGG | 0.935 |
| rpl16 | 104 | 35 | ACU->AUU | Thr->Ile | TGGTTTTGGAAGATATGGCA | C | TAAAAGTTGTAGAGCTGGTC | 1 |
| rpl16 | 164 | 55 | ACA->AUA | Thr->Ile | TGAAGCAGCGCGTCGGGCTA | C | AATCGGACAATTCCATCGTG | 1 |
| rps12 | 71 | 24 | UCG->UUG | Ser->Leu | CACGGACCGTACTCGAGCTT | C | GGATCAATGTCCCCAGAAGC | 1 |
| rps12 | 100 | 34 | CGC->UGC | Arg->Cys | GTCCCCAGAAGCAAGGAGTA | C | GCCCGCGTGTTTCAACGAGA | 0.999 |
| rps12 | 104 | 35 | CCG->CUG | Pro->Leu | CCAGAAGCAAGGAGTACGCC | C | GCGTGTTTCAACGAGAACAC | 1 |
| rps12 | 146 | 49 | CCA->CUA | Pro->Leu | GAAAAAACCTAATTCAGCTC | C | ACGTAAGATAGCCAAAGTAC | 1 |
| rps12 | 196 | 66 | CAC->UAC | His->Tyr | ATCGACATGATATATTTGCT | C | ACATTCCGGGCGAAGGTCAT | 0.998 |
| rps12 | 221 | 74 | UCG->UUG | Ser->Leu | TCCGGGCGAAGGTCATAATT | C | GCAGGAACATTCTATGGTCT | 0.999 |
| rps12 | 284 | 95 | UCC->UUC | Ser->Phe | AGATTTGCCAGGTGTGAAAT | C | CCATTGTATTCGAGGAGTCA | 0.999 |
| rps13 | 5 | 2 | UCA->UUA | Ser->Leu | AAAGGAAGGATCAAGAATGT | C | ATATATTTCAGGAGCTAGAT | 0.991 |
| rps13 | 26 | 9 | UCA->UUA | Ser->Leu | ATATATTTCAGGAGCTAGAT | C | AGTTGCCGATGAACAAGTAA | 0.999 |
| rps13 | 56 | 19 | UCA->UUA | Ser->Leu | TGAACAAGTAAGAATTGCCT | C | AACAAAAATTGATGGAATTG | 0.996 |
| rps13 | 100 | 34 | CGU->UGU | Arg->Cys | CTAAAAAAGCCATTCAGGTT | C | GTTATCGATTAGGTATCAGT | 0.993 |
| rps4 | 43 | 15 | CGG->UGG | Arg->Trp | GTCTACTTTCAGGAAATGTT | C | GGAAAAGAGAACTTACAATA | 0.999 |
| rps4 | 127 | 43 | CCG->UCG | Pro->Ser | CTATTAAGAGAAAGATTTAT | C | CGAGAGAAAATCTTAACAGT | 0.998 |
| rps4 | 158 | 53 | UCA->UUA | Ser->Leu | TCTTAACAGTTACATCCAAT | C | ACAAACTACACGAAAGTTGC | 0.996 |
| rps4 | 178 | 60 | CCC->UCC | Pro->Ser | CACAAACTACACGAAAGTTG | C | CCCTTTTTCATGGGGATTTA | 1 |
| rps4 | 187 | 63 | CAU->UAU | His->Tyr | CACGAAAGTTGCCCCTTTTT | C | ATGGGGATTTACCGCACAGA | 0.986 |
| rps4 | 239 | 80 | CCA->CUA | Pro->Leu | AACTTCCTATATCCCTTTTC | C | ACTCAATCCAGAAACAAGAT | 1 |
| rps4 | 248 | 83 | CCA->CUA | Pro->Leu | TATCCCTTTTCCACTCAATC | C | AGAAACAAGATCGGACGTTA | 0.972 |
| rps4 | 260 | 87 | UCG->UUG | Ser->Leu | ACTCAATCCAGAAACAAGAT | C | GGACGTTATTCCGGTTCGTC | 0.997 |
| rps4 | 272 | 91 | CCG->CUG | Pro->Leu | AACAAGATCGGACGTTATTC | C | GGTTCGTCTCCATTTTTGTG | 0.998 |
| rps4 | 317 | 106 | CCG->CUG | Pro->Leu | TATTCCTCAAGCAAGGCAGC | C | GATAAGTCATCGAAGGGTTT | 0.999 |
| rps4 | 464 | 155 | UCA->UUA | Ser->Leu | ATCTTTCTATATCGAAATAT | C | AGTTGAAAAAATCATAGGCA | 0.998 |
| rps4 | 902 | 301 | UCG->UUG | Ser->Leu | TGAACTACCTACTCATTATT | C | GGAGGTGAATCATAGAACAC | 0.995 |
| rps4 | 913 | 305 | CAU->UAU | His->Tyr | CTCATTATTCGGAGGTGAAT | C | ATAGAACACCAAAAGCTGTG | 0.991 |
| rps4 | 923 | 308 | CCA->CUA | Pro->Leu | GGAGGTGAATCATAGAACAC | C | AAAAGCTGTGGTATCTTATG | 1 |
| rps4 | 938 | 313 | UCU->UUU | Ser->Phe | AACACCAAAAGCTGTGGTAT | C | TTATGGACCTAACATAGGTC | 0.999 |
| rps4 | 1003 | 335 | CGG->UGG | Arg->Trp | AAGATCTAAACCTTCTTCTT | C | GGAGCGGAAACGGACGTGGT | 0.997 |
| rps7 | 116 | 39 | CCA->CUA | Pro->Leu | TTATCAAACTTTTCATCGCC | C | AGCTCAAACTGAACGCGATG | 0.951 |
| rps7 | 332 | 111 | UCA->UUA | Ser->Leu | GATAAGCTTAGAGAAATGTT | C | ATTTGCTGAAATACTGGATG | 0.984 |
| sdh3 | 45 | 15 | CUC->CUU | Leu->Leu | CCTATTTATAAGCCACAGCT | C | ACTTCGACGTTTCCAATTGC | 0.989 |
| sdh3 | 58 | 20 | CCA->UCA | Pro->Ser | CACAGCTCACTTCGACGTTT | C | CAATTGCCAATAGAATATCA | 0.998 |
| sdh4 | 122 | 41 | ACC->AUC | Thr->Ile | AAGCTCGAAGACAAAGAGAA | C | CGGGCTTTTCCAAAGAATTA | 0.973 |
